# Host-adaptive traits in the plant-colonizing *Pseudomonas donghuensis* P482 revealed by transcriptomic responses to exudates of tomato and maize

**DOI:** 10.1038/s41598-023-36494-6

**Published:** 2023-06-09

**Authors:** Dorota M. Krzyżanowska, Magdalena Jabłońska, Zbigniew Kaczyński, Małgorzata Czerwicka-Pach, Katarzyna Macur, Sylwia Jafra

**Affiliations:** 1 Laboratory of Plant Microbiology, Intercollegiate Faculty of Biotechnology UG and MUG, University of Gdańsk, ul. A. Abrahama 58, 80-307 Gdańsk, Poland; 2 Laboratory of Structural Biochemistry, Faculty of Chemistry, University of Gdańsk, ul. Wita Stwosza 63, 80-308 Gdańsk, Poland; 3 Laboratory of Mass Spectrometry, Intercollegiate Faculty of Biotechnology UG and MUG, University of Gdańsk, ul. A. Abrahama 58, 80-307 Gdańsk, Poland

**Keywords:** Microbiology, Bacteria, Bacteriology, Microbial genetics

## Abstract

Pseudomonads are metabolically flexible and can thrive on different plant hosts. However, the metabolic adaptations required for host promiscuity are unknown. Here, we addressed this knowledge gap by employing RNAseq and comparing transcriptomic responses of *Pseudomonas donghuensis* P482 to root exudates of two plant hosts: tomato and maize. Our main goal was to identify the differences and the common points between these two responses. Pathways upregulated only by tomato exudates included nitric oxide detoxification, repair of iron-sulfur clusters, respiration through the cyanide-insensitive cytochrome *bd*, and catabolism of amino and/or fatty acids. The first two indicate the presence of NO donors in the exudates of the test plants. Maize specifically induced the activity of MexE RND-type efflux pump and copper tolerance. Genes associated with motility were induced by maize but repressed by tomato. The shared response to exudates seemed to be affected both by compounds originating from the plants and those from their growth environment: arsenic resistance and bacterioferritin synthesis were upregulated, while sulfur assimilation, sensing of ferric citrate and/or other iron carriers, heme acquisition, and transport of polar amino acids were downregulated. Our results provide directions to explore mechanisms of host adaptation in plant-associated microorganisms.

## Introduction

Plants nourish microbial communities in the rhizosphere by releasing blends of organic compounds through the roots^1^. Root exudates contain primary metabolites such as organic acids, amino acids, sugars and secondary metabolites with bioactive or signalling properties. The exact chemical composition of exudates depends on the plant species and the plant's physiological status, the latter dependent on the developmental stage, nutrient availability, and the presence of stressors^2^. Differences in the composition of exudates and the workings of the plant's innate immunity shape the composition and the activity of the root microbiota^3^.

*Pseudomonas* bacteria can thrive in various environmental niches, including the roots of various plant hosts. Their competitive edge involves metabolic flexibility and producing a wide range of secondary metabolites, including antimicrobials and iron-scavenging compounds^4^. Many plant-associated strains facilitate plant growth, alleviate abiotic stress, or protect plants against pathogens^5^. There are no comprehensive studies of the phylogenetic breadth of plants that a given *Pseudomonas* strain can colonize. However, certain pseudomonads have proven to be effective as biocontrol agents on plant species different from that of their origin or on multiple crops, suggesting that pseudomonads are rather promiscuous colonists of plants^6^.

There is growing recognition that plant species select for distinctive microbial communities^7^, with more phylogenetically distant plant hosts recruiting the most distinct microbiota populations^8^. Therefore, the apparent host promiscuity of some microorganisms, such as pseudomonads, raises questions on the metabolic changes required of bacteria to colonize multiple hosts or to maintain an association with a host that undergoes physiological changes. This issue has been hard to address with existing data since most studies address only single host-microbe interactions. Furthermore, while determinants of host specificity in plant–microbe interactions have been studied in depth for symbiotic rhizobia, it has received little attention in bacteria that form less intimate associations with their hosts^9^.

*Pseudomonas donghuensis* P482 is a biocontrol strain that inhibits the growth of several bacterial and fungal plant pathogens^10,11^. Originally isolated from the rhizosphere of tomato (*Solanum lycopersicum* L.), the bacterium can also colonize the rhizosphere of potato^12^ and, as shown in this study, the roots of maize, altogether making it a promising model for studying host-adaptive traits in promiscuous root colonizing bacteria.

In this work, we investigate which metabolic pathways can be a part of an adaptive response of pseudomonads to different plant hosts by identifying genes of differentiating ('host-specific') and the shared ('host-independent') transcriptomic responses of *Pseudomonas donghuensis* P482 to root exudates of two phylogenetically distinct plant species: tomato (Dicot) and maize (Monocot).

## Materials and methods

The methods of this study are in accordance with relevant institutional, national, and international guidelines and legislation. This study protocols also complies with the IUCN Policy Statement on Research Involving Species at Risk of Extinction and the Convention on the Trade in Endangered Species of Wild Fauna and Flora.

### Colonization of the rhizosphere of tomato and maize

The rhizosphere-colonization assay was performed using P482 Rif, a spontaneous rifampicin-resistant mutant of P482^12^. Plants were grown for 18 days in non-sterile soil from P482-inoculated seeds. Experimental details can be found in Supplementary Information (SI).

### Plant material

Tomato (*Solanum lycopersicum* L.) cv. Saint Pierre (Vilmorin Garden, Poland) and maize (*Zea mays* L.) cv. Bejm (provided by the Plant Breeding and Acclimatization Institute, Smolice, Poland) were grown from seed.

### Plant growth in gnotobiotic conditions

Tomato seeds were sterilized by treating with 70% ethanol for 1 min, followed by 3 min in 3% NaOCl and 3 times rinsing in sterile distilled water. Seeds of maize were surface sterilized by treating twice with 3% NaOCl for 15 min, followed by 10 min in 70% ethanol and 3 times rinsing with sterile distilled water. Surface-sterilized seeds were germinated on Germination Medium (GM) (0.5 × Murashige and Skoog Medium with Gamborg B5 Vitamins, 2% sucrose, 0.2% wheat peptone and 0.7% of plant agar, pH ≈ 6.1). The advantage of using GM is that, thanks to the addition of peptone, it enables screening of the germinating seeds for microbial contamination. Tomato was germinated in the dark at 24 °C, while maize was in light at 22 °C. Germinated seeds with no sign of infection were transferred to containers with large-grain quarts sand (1.4–2 mm) (AQUAEL, Poland). Culture conditions were optimized for the size and nutritional requirements of each species: maize was cultured in 900 mL glass jars in ¼ Hoagland's medium, pH 5.6–5.8 (Hoagland's No. 2 Basal Salt Mixture, Merck), 3 seedlings per jar, while tomato was cultured in GA-7 Magenta vessels (Merck), 6 plants per vessel, in ½ Hoagland's medium. The plants were grown for 13 days at 22 °C, 16 h light/8 h dark photoperiod. Both plant species established a second pair of leaves within this period.

### Collection of root exudates

Plants grown in gnotobiotic conditions were removed from the sand. Roots were washed in water for 2–4 min, transferred to glass beakers containing high-purity water sterile water, and incubated for 2 h. Some leaves of the small tomato plants touched the water surface. Exudates from 174 tomato plants and 48 maize plants were collected to obtain enough exudates for bacterial cultures (RNAseq) and chemical analyses. This number of plants yielded 4.5 mg of tomato exudates and 6.6 mg of maize exudates. The samples from each species were pooled and filter-sterilized through a 0.22 µm low-binding PES membrane (Thermo Scientific). The samples were frozen at − 80 °C and freeze-dried.

### Chemical analysis of the composition of root exudates

Non-targeted analysis of the chemical composition of root exudates was performed using gas chromatography-mass spectrometry (GC–MS) and nuclear magnetic resonance (NMR). The relative quantity of 21 amino acids was assessed with liquid chromatography-selected reaction monitoring mass spectrometry (LC-SRM). All details are provided in Supplementary Information (SI).

### Growth of P482 in the presence of root exudates

P482 was grown at 28 °C in 1C medium (M9 salts, 2 mM of Mg_2_SO_4_, 0.1 mM CaCl_2,_ 0.4% glucose^13^), with or without (control) freeze-dried exudates of tomato or maize. The experiment was performed in 96-well plates, incubated with orbital shaking in an EPOCH microplate reader (BioTek), with OD_600_ measured every 20 min. Three concentrations of exudates (0.02 mg L^−1^, 0.1 mg L^−1^, 0.2 mg L^−1^) were tested to verify their impact on the growth parameters of P482 (Fig. S1). The highest concentration was later applied to grow cells for interrogation with RNAseq since the highest concentration was deemed most likely to result in exudate-induced changes in gene expression. The pH of the media was measured before inoculation and following the growth of P482 to an early stationary phase using MQuant pH indicator strips (Supelco).

### Harvest of bacterial cells and RNA extraction

Bacteria were harvested upon reaching the early stationary phase, following 33 h of growth in unsupplemented 1C medium and 24 and 28 h, respectively, for the growth in 1C medium with maize or tomato exudates (OD_600_ 0.35–0.48) (Fig. S1). Per each treatment, three pools of cells (ca. 10^9^) were collected by centrifugation and fixed with fixRNA (Eurx, Poland). Each pool was treated as a separate biological replicate in the downstream RNAseq. Total RNA was isolated using RNeasy Mini Kit (Qiagen). Purified RNA was treated with TURBO DNA-free™ Kit (Thermo Fisher Scientific). The lack of gDNA contamination was confirmed by real-time PCR with primers targeting *gyrB*.

### RNAseq and data analysis

Nine RNA samples were sequenced, with 3 biological replicates per each of the two exudate treatments and the control (1 C medium alone). Assessment of RNA integrity, depletion of rRNA, library preparation, Illumina sequencing (min. 5 GB per sample), transcriptome assembly, and differential gene expression analysis was outsourced to Baseclear (Leiden, The Netherlands). Filtered RNA-seq reads were mapped to the reference genome of P482 (Genbank: JHTS00000000.1)^14^. Statistics following filtering and alignment are available in Tables S1 and S2, respectively. Principal component analysis was applied to verify whether the replicate samples form separate clusters (Fig. S2). Differential gene expression analysis was performed using DESeq2 1.22.2^15^. Further analyses were performed in-house. The changes in gene expression were filtered for biological (1.5 log_2_ fold change (FC)) and statistical significance (*p* < 0.05; adjusted *p* value, Benjamini–Hochberg (B–H) correction to control false discovery rate (FDR)). Genes with *p*adj > 0.05 and those that could not been assigned the adjusted value (NA) were excluded form downstream analysis. Overlapping groups of differentially-expressed genes were visualized with BioVenn^16^. Proteins were assigned to Clusters of Orthologous Groups (COGs) using eggNOG mapper 5.0^17^ and to KEGG metabolic pathways using BlastKOALA^18–21^. Enrichment within COGs and KEGG pathways was established using the genome of P482 as a reference (JHTS00000000.1), with Fisher's exact test applied to determine the statistical significance (*p* < 0.05; adjusted *p* value, B–H correction). Gene networking and cluster enrichment were analyzed using STRING 11.5 (May 2023)^22^, with the genome of *P. donghuensis* HYS as a reference^11^.

### cDNA synthesis and real-time RT-qPCR

RNAseq data were validated by RT-qPCR using six targets with different expression patterns: BV82_3254, *mexE*, *norC*, *ssuC*, *trpB,* and *ytfE*. Reverse transcription and real-time qPCR were performed as described earlier^23^. Primer details are available in Table S3. The size of the amplicons was confirmed by gel electrophoresis (Fig. S3). Gene expression analysis was performed using qbase + ^24^. The applied reference genes, *gyrB,* and *rpoD,* were chosen based on our previous work^23^ and confirmed for stability in the analyzed dataset (average M = 0.543; CV = 0.181). Expression was scaled to the samples originating from P482 grown in 1C medium without exudates (untreated). The statistical significance of differences in expression was evaluated using an unpaired t-test.

## Results and discussion

### *P. donghuensis* P482 achieved high populations on the roots of soil-grown tomato and maize, confirming it to be a suitable model for studying bacterial adaptations to different hosts

P482 Rif was present on the roots of 18-day-old tomato and maize plants grown in soil from inoculated seeds. Based on arithmetic means, the population size on tomato roots was approximately tenfold higher than that on the roots of maize, amounting 1.54 × 10^7^ CFU g^−1^ on tomato and 2.35 × 10^6^ CFU g^−1^ on maize (Fig. S4). When median values were considered, the difference was 25-fold, with population sizes of 8.1 × 10^6^ CFU g^−1^ and 3.1 × 10^5^ CFU g^−1^ for tomato and maize, respectively. Despite the difference, the population sizes of P482 on both plants can be considered high, implying that the strain can adapt to colonize both hosts.

### Exudates of tomato caused more extensive changes in the transcriptome of P482 than the exudates of maize

Whole transcriptome profiling was performed for P482 cells grown in the presence of root exudates of either tomato or maize (0.2 mg L^−1^), both of which stimulated the growth of the bacterium compared to 1C medium alone (Fig. S1A, B). 5168 genes were detected in the transcriptome, covering 99% of the entire genome. The reliability of measuring the abundance of transcripts by RNAseq was confirmed using reverse transcription real-time qPCR (RT-qPCR) for selected targets (Fig. S5).

**Figure 1:**
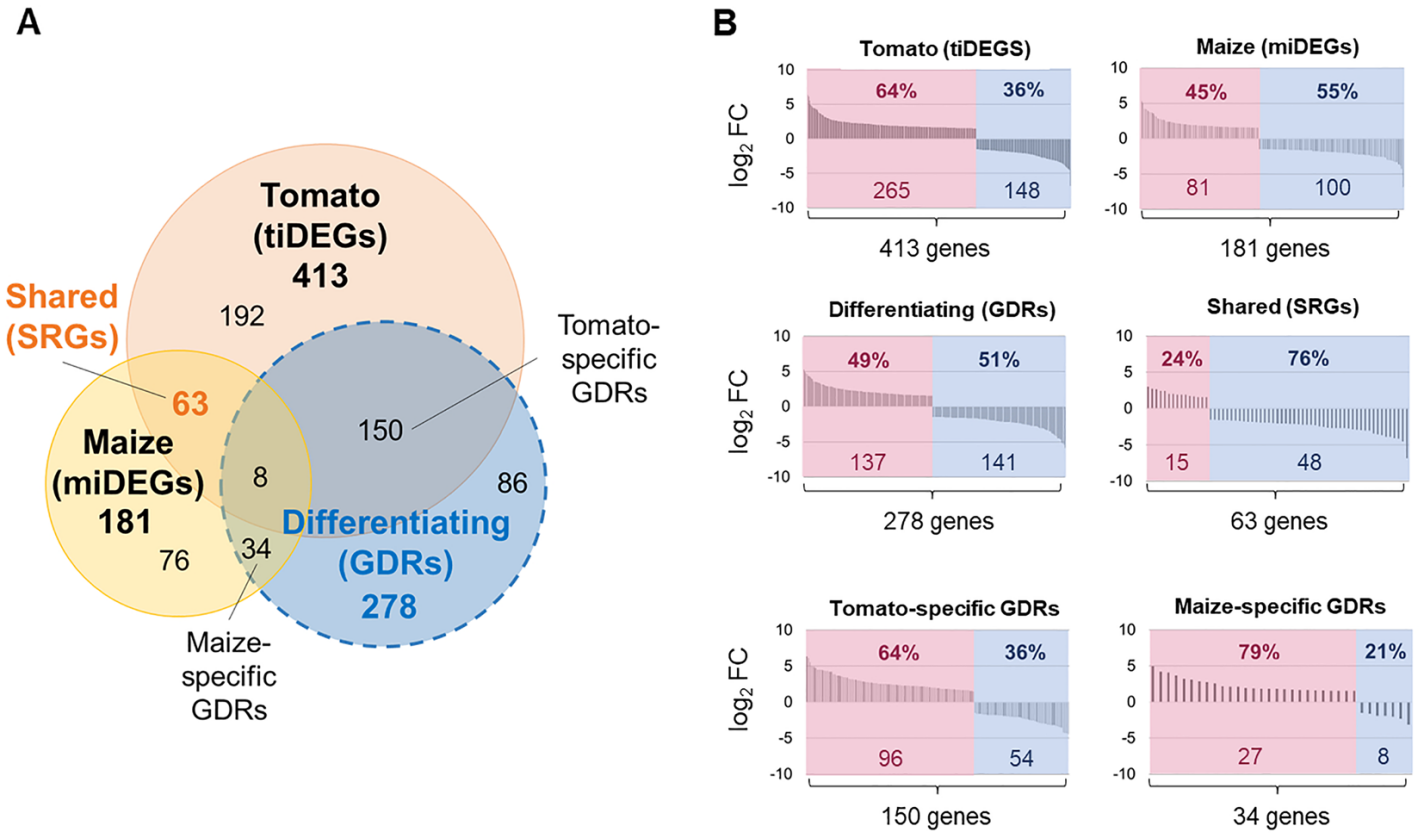
Venn diagram depicting the subsets of genes in the tomato- and maize-triggered transcriptomes of *P. donghuensis* P482 (**A**), and the proportion of genes up- and downregulated in each subset (**B**). tiDEGs (413) and miDEGs (181) showed significantly different expression in response to the root exudates of either tomato or maize, respectively, when compared to unsupplemented medium (control). Contrary, the genes of the differentiating response (GDRs, 278), a set encircled by a dotted line, showed a significant change in expression between the two exudate treatments. The shared response genes (SRGs, 63) were established by superimposition of miDEGs, tiDEGs and GDRs, and comprise genes affected by both types of exudates in a similar manner. Additional subsets resulting from the superimposition are tomato-specific GDRs (150) and maize-specific GDRs (34). Those concomitantly met two criteria: they were among GDRs and at the same time their expression was significantly different then in the medium when treated with only one exudate type. Significance cut-offs for a significant change in expression: *p* < 0.05 (padj; B–H correction); log_2_FC > 1.5. A list of loci in each subset can be found in Dataset S6. In panel B, upregulated genes are shown in magenta (on the left) and downregulated genes are in blue (on the right). Both the percentage of genes and the actual ORF count are indicated in the graphs.

Tomato exudates altered the expression of 413 genes (7.99%) compared to the unsupplemented medium. These are further referred to as tomato-induced Differentially Expressed Genes (tiDEGs). Maize exudates influenced the expression of 181 genes (3.51%), further referred to as miDEGs (maize-induced DEGs) (Fig. 1). The fact that there were more tiDEGs than miDEGS, and that they had a higher mean log_2_FC value (2.27 log_2_FC vs. 2.14 log_2_FC; Dataset S1), suggests that the response of P482 to the exudates of tomato was broader and more pronounced than that to the exudates of maize. It is an open question whether this is related to the fact that maize is not the original host for P482. A similar scale of transcriptomic response as for P482 and tomato was reported for *P. aeruginosa* PAO1 growing in CAA medium supplemented with the exudates of beetroot (*Beta vulgaris*)^25^. In the latter study, aimed at identifying novel and presumably host-specific genes involved in plant–microbe interactions, the authors compared the response of PAO1 to exudates of two beetroot varieties, Celt and Roberta, known to select for different microbial communities. Depending on the variety, 9.30% and 8.13% of the transcriptome of PAO1 was affected by exudates.

### More genes were affected by exudates in a host-specific manner than as a part of a shared (host-independent) response

Our aim in this study was to identify metabolic pathways that are: (1) differentially and (2) similarly regulated in P482 in response to the exudates of tomato and maize. To do that, we determined two gene pools within the transcriptome: Genes of the Differentiating Response (GDRs) and the Shared Response Genes (SRGs). GDRs (278 genes) are genes with expression significantly different between the two exudate treatments (Fig. 1; Dataset S2). On the contrary, SGRs (63 genes) show similar responses to exudates independent of the source plant. The superimposition of GDRs, tiDEGs, and miDEGs determined the pool of SGRs. This analysis additionally enabled the distinguishing of subsets of GDRs which we termed tomato-specific GDRs (150 genes) and maize-specific GDRs (34 genes) (Fig. 1; Dataset S2). Genes in the plant-specific GDR subsets concomitantly met two criteria: they showed different expression upon the two exudate treatments (> 1.5 log_2_FC, padj < 0.05), making them GDRs, but also their expression was significantly different (> 1.5 log_2_FC, padj < 0.05) compared to 1C medium upon treatment with only one of the exudates. The tomato-specific and maize-specific GDRs, along with SGRs, were analyzed for top-ranking up- and down-regulated genes (Tables S4–S6). Establishing plant-specific GDRs helped us to assign certain aspects of overall differentiating response to one of the plants. It also prevented the underreporting of maize-driven aspects of the differentiating response, considering that tomato exudates, with a more significant share of GDRs, are the dominant driver of the overall changes.

**Figure 2:**
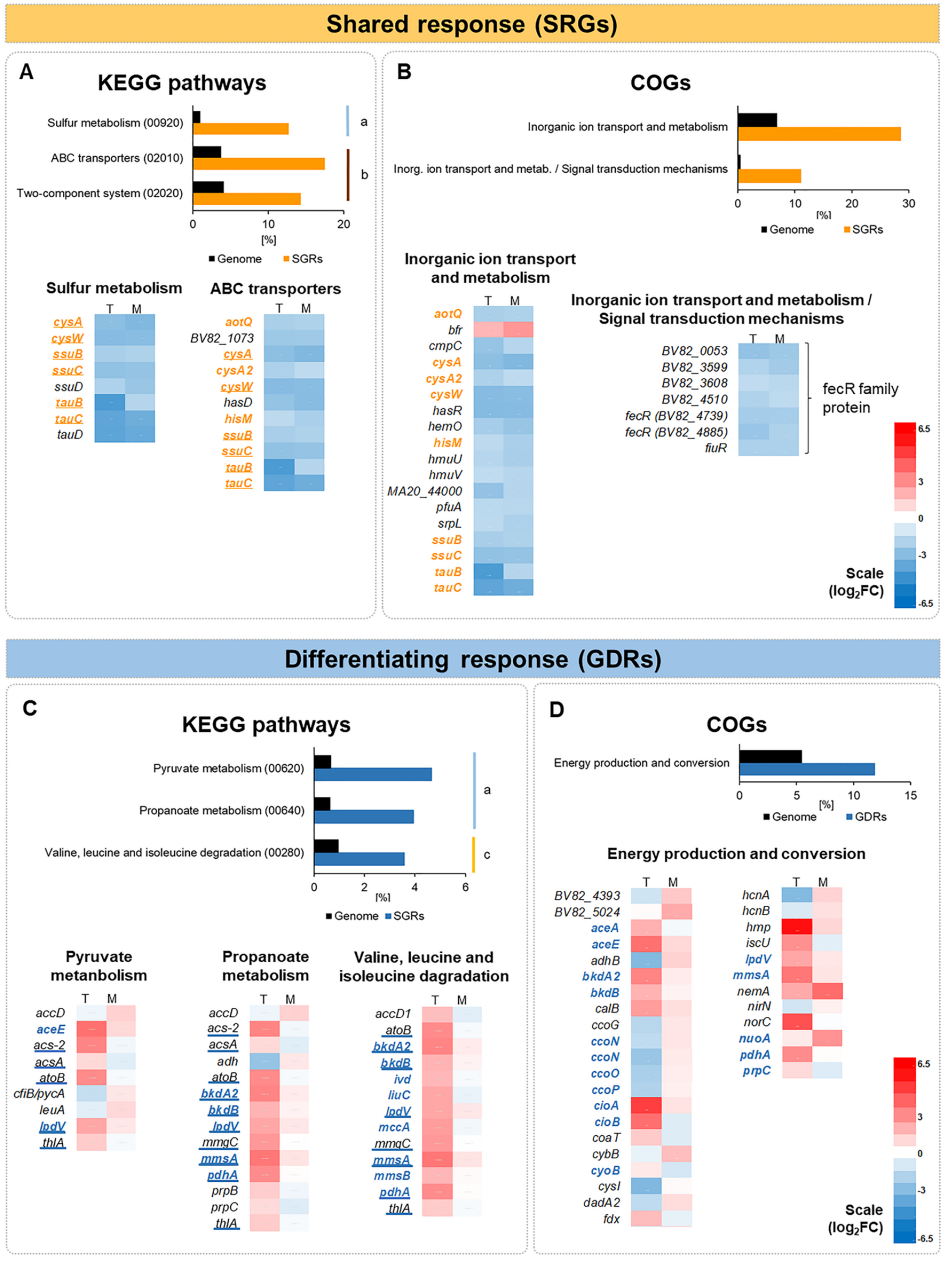
KEGG pathways and COG categories significantly enriched within the shared (**A, B**) and differentiating (**C, D**) transcriptomic responses of P482 to tomato and maize root exudates. For individual genes within pathways: red and blue indicate upregulation and downregulation of gene expression, respectively; the left column shows changes in expression (log_2_FC) upon the addition of tomato exudates (letter T), and the right column upon the addition of maize exudated (letter M). KEGG categories: a—carbohydrate metabolism, b—environmental information processing, c—amino acid metabolism. Genes shown in bold color font are listed within enriched KEGG pathways and COGs. Underlined genes overlap between categories within the KEGG pathways.

**Figure 3:**
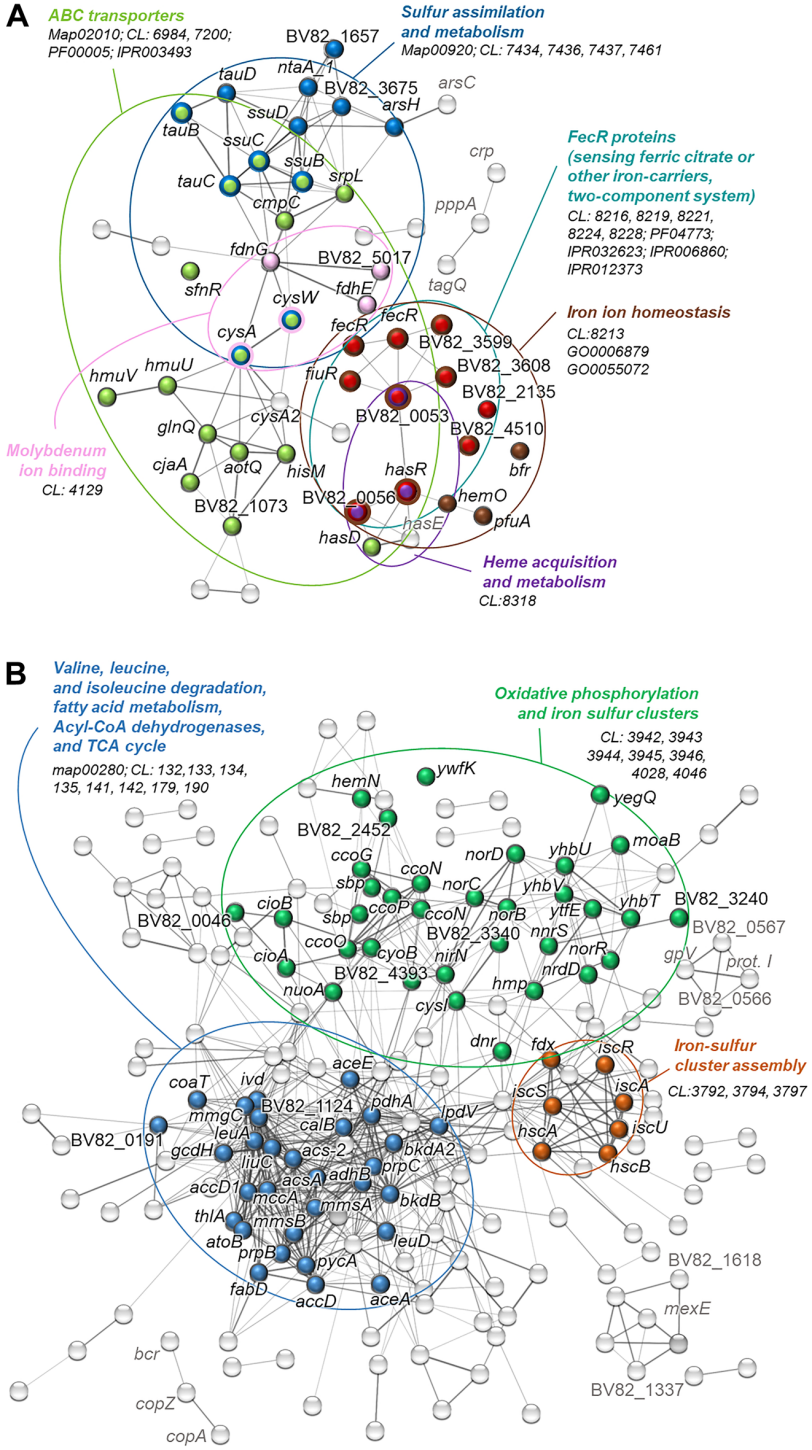
Gene networking for the 63 genes of the shared (**A**) and 278 genes of the differentiating (**B**) transcriptomic response of P482 to plant root exudates. The analysis was performed using STRING with the genome of *P. donghuensis* HYS as reference, and the image was manually curated. Each node represents a gene of P482 with a name assigned with eggNOG. Filled-in nodes mark genes that belong to enriched groups identified by STRING (map—KEGG pathway, CL—cluster, GO—gene ontology, PF, IPR—protein domains). Network edges indicate confidence. Interaction based on all available sources for version 11.5 (May 2023). Minimal interaction score 0.4 (medium). Disconnected nodes were disabled, except for genes that were found to be a part of enriched clusters. Each gene can be matched with a locus designation using Dataset S5. Full list of STRING interactions and the enriched groups is provided in Dataset S4.

The complete pool of 278 GDRs (differentiating response) and 63 SGRs (shared response) were analyzed for KEGG and COG enrichment and gene networking (Figs. 2, 3; Datasets S3–S4). Most interesting findings are presented and discussed below, while a few minor pathways are described in SI.

GDRs accounted for 5.38% of the transcriptome, with an approximately even share of downregulated and upregulated. In comparison, the SGRs accounted for 1.22% of the transcriptome, most of them downregulated (76%) (Fig. 1). Therefore, the pool of genes differently affected by two hosts in P482 was approx. 4.5-times greater than the pool of genes of the shared response. The same was observed for PAO1 in two beetroot varieties^25^. In the case of distinct plant hosts such as tomato and maize, it is tempting to attribute this effect to the overall species-related differences in the composition of exudates. However, the fact that a similar tendency was observed for two varieties of a single plant species, the beetroot, suggests that relatively minor changes in the composition of plant-derived compounds may have a game-changing impact. In line with this assumption, compounds such as benzoxazinoids and coumarins have been shown to single-handedly exert a profound effect on the composition of root-associated microbiota^26,27^.

### Host-independent response to exudates

#### Sulfur assimilation

Both exudate treatments caused the downregulation of P482 genes within the overlapping pathways for 'Sulphur metabolism' and 'ABC transporters' (Fig. 2a, Table 1). This included genes encoding the CysW and CysA components of the sulfate-thiosulphate permease (SulT). Sulphate and thiosulphate represent inorganic sources of sulfur utilized by bacteria^28^. In the absence of inorganic sulfate or cysteine, bacteria can use alkanesulfonates, including taurine, as alternative sulfur sources^28^. In P482, both exudates downregulated the expression of *tauBC* and *ssuBC*, involved in the import of taurine and other alkanesulfonates. A similar influence was observed for *ssuD* and *tauD*, involved in releasing sulfur from those compounds^29^. Additionally, STRING enabled the identification of another downregulated gene possibly involved in sulfur acquisition in P482, the *sfnR* (Fig. 3a). In *Pseudomonas putida* DS1, *sfnR* was found to be necessary for obtaining sulfur from dimethyl sulfide (DMS)^30^.

**Table 1:** Selected genes of the shared and differentiating responses of P482 to plant exudates.

Locus	Gene	Predicted product/function	log_2_FC^A^	
			Tomato	Maize
*Shared response*				
*Sulphur assimilation*				
BV82_1667	*tauC*	Binding–dependent transport system inner membrane component f.p	− 4.05	− 3.65
BV82_1668	*tauB*	ABC transporter family protein	− 4.52	− 1.87
BV82_1666	*tauD*	Alpha-ketoglutarate-dependent taurine dioxygenase	− 4.11	− 3.83
BV82_1677	*ssuB*	Aliphatic sulphonates import ATP-binding protein SsuB	− 2.22	− 2.02
BV82_1676	*ssuC*	Binding–dependent transport system inner membrane component f.p	− 2.79	− 2.72
BV82_1675	*ssuD*	Alkane sulphonate monooxygenase, FMNH(2)-dependent	− 2.04	− 2.57
BV82_2456	*cysA*	Sulphate ABC transporter, ATP-binding family protein	− 2.89	− 3.19
BV82_2499	*cysA2*	Putative 2-aminoethylphosphonate ABC transporter, ATP-bind. prot	− 2.07	− 1.66
BV82_2455	*cysW*	Sulphate ABC transporter, permease protein CysW	− 3.05	− 2.98
BV82_3639	*sfnR*	AAA domain family protein	− 3.42	− 2.34
*Arsenic resistance*				
BV82_3873	*arsC*	Low mol. weight phosphotyrosine phosphatase family protein	2.42	2.66
BV82_3874	*arsH*	Arsenical resistance protein ArsH	2.63	2.35
*Sensing of ferric citrate and/or xenosiderophores and iron homeostasis*				
BV82_0053		FecR family protein	− 2.73	− 2.49
BV82_1854	*fiuR*	FecR family protein	− 2.09	− 2.06
BV82_3599		FecR family protein	− 1.96	− 2.33
BV82_3608		FecR family protein	− 1.59	− 1.78
BV82_4510		FecR family protein	− 2.00	− 1.67
BV82_4739	*fecR*	FecR family protein	− 2.29	− 2.25
BV82_4885	*fecR*	FecR family protein	− 2.57	− 2.02
BV82_4419	*bfr*	Bacterioferritin	1.87	2.67
*Haem acquisition and metabolism*				
BV82_0055	*hasR*	HasR protein	− 3.09	− 3.05
BV82_0056		Haem-binding A family protein	− 6.83	− 6.84
BV82_0057	*hasD*	Type I secretion system ATPase family protein	− 2.71	− 3.03
BV82_0058	*hasE*	Type I secretion membrane fusion, HlyD family protein	− 3.87	− 3.32
BV82_0378	*hemO*	Haem oxygenase family protein	− 2.25	− 2.60
BV82_4822	*hmuV*	ABC transporter family protein	− 1.63	− 1.86
BV82_4823	*hmuU*	fecCD transport family protein	− 1.86	− 2.20
*Regulatory pathway for T6SS assembly*				
BV82_1739	*pppA*	Phosphatase 2C family protein	1.89	1.86
BV82_1744	*tagQ*	Glycine-zipper containing OmpA-like membrane domain protein	2.03	1.54
BV82_1931	*crp*	Cyclic AMP receptor-like protein	2.03	2.05
*Differentiating response*				
*Nitric oxide detoxification*				
BV82_3238	*norR*	AAA domain family protein	− 1.67	1.41
BV82_3241	*nnrS*	nnrS family protein	4.99	0.32
BV82_3242	*dnr*	Crp/Fnr family transcriptional regulator	3.23	0.31
BV82_3244	*norD*	von Willebrand factor type A domain protein	4.19	0.60
BV82_3245	*norB*	Nitric oxide reductase subunit B	4.18	0.31
BV82_3246	*norC*	Nitric oxide reductase subunit C	4.44	− 0.07
BV82_4743	*hmp*	Oxidoreductase NAD-binding domain protein	6.13	1.05
*Iron sulphur clusters biogenesis and repair*				
BV82_0252	*iscR*	rrf2 family protein	2.40	− 0.09
BV82_0253	*iscS*	Cysteine desulfurase IscS	2.67	− 0.28
BV82_0254	*iscU*	fe-S cluster assembly scaffold IscU	2.84	− 0.71
BV82_0255	*iscA*	Iron-sulphur cluster assembly protein IscA	2.97	− 0.84
BV82_0256	*hscB*	fe-S protein assembly co-chaperone HscB	2.29	− 0.89
BV82_0257	*hscA*	fe-S protein assembly chaperone HscA	1.76	− 0.79
BV82_0258	*fdx*	Ferredoxin, 2Fe-2S type, ISC system	1.72	− 0.63
BV82_3239	*ytfE*	Hemerythrin HHE cation binding domain protein	6.36	0.48
*Terminal oxidases and respiration*				
BV82_0044	*cioA*	Bacterial Cytochrome Ubiquinol Oxidase family protein	4.92	0.73
BV82_0045	*cioB*	Cytochrome d ubiquinol oxidase, subunit II	3.67	− 0.83
BV82_0999	*ccoP*	Cytochrome c oxidase, cbb3-type, subunit III	− 2.01	0.43
BV82_1000	*ccoQ*	cbb3-type cytochrome oxidase component FixQ family protein	− 3.1	0.05
BV82_1001	*ccoO*	Cytochrome c oxidase, cbb3-type, subunit II	− 2.16	0.47
BV82_1002	*ccoN*	Cytochrome c oxidase, cbb3-type, subunit I	− 1.63	0.68
BV82_1198	*ccoG*	Cytochrome c oxidase accessory protein CcoG	− 1.8	0.64
BV82_1235	*nuoA*	NADH-ubiquinone/plastoquinone oxidoreductase	0.54	2.77
BV82_4001	*cybB*	Prokaryotic cytochrome b561 family protein	− 0.57	1.68
BV82_5034A	*ccoN*	Cytochrome C and Quinol oxidase polypeptide I family protein	− 2.56	0.55
*Catabolism of amino acids/fatty acids and the glyoxylate shunt*				
BV82_0008	*mmsA*	Methylmalonate-semialdehyde dehydrogenase	3.49	0.71
BV82_0009	*mmsB*	3-Hydroxyisobutyrate dehydrogenase	1.87	0.15
BV82_0366	*gcvP*	Glycine dehydrogenase	1.79	− 0.21
BV82_0367	*gcvH*	Glycine cleavage system H protein	1.19	− 1.63
BV82_0838	*lpdV*	Dihydrolipoyl dehydrogenase	2.26	0.63
BV82_0839	*bkdB*	Lipoamide acyltransferase component of branched-chain alpha-keto acid dehydrogenase complex	1.95	0.34
BV82_0840	*bkdA2*	2-Oxoisovalerate dehydrogenase subunit beta	3.14	0.52
BV82_0841	*pdhA*	2-Oxoisovalerate dehydrogenase subunit alpha	2.94	0.10
BV82_1123	*acs-2*	AMP-binding enzyme family protein	3.12	− 0.35
BV82_1125	*atoB*	Acetyl-CoA C-acetyltransferase family protein	2.97	− 0.19
BV82_1126	*mmgC*	Acyl-CoA dehydrogenase, N-terminal domain protein	2.64	− 0.01
BV82_1237	*aceA*	Isocitrate lyase	2.00	− 0.27
BV82_2129	*ivd*	Acyl-CoA dehydrogenase, N-terminal domain protein	1.86	− 0.19
BV82_2130	*accD1*	Carboxyl transferase domain protein	1.68	− 0.71
BV82_2131	*liuC*	Enoyl-CoA hydratase/isomerase family protein	2.27	− 0.52
BV82_2132	*mccA*	hlyD secretion family protein	2.53	− 0.32
BV82_4665	*aceE/mdeB*	Pyruvate dehydrogenase (acetyl-transferring), homodimeric type	3.65	1.01
BV82_5069	*adhB*	Iron-containing alcohol dehydrogenase family protein	− 3.12	1.17
BV82_5184	*adh*	Zinc-binding dehydrogenase family protein	− 2.68	0.63
BV82_0795	*phhC*	Aminotransferase class I and II family protein	3.58	0.72
BV82_0796	*phhB*	Putative pterin-4-alpha-carbinolamine dehydratase	4.36	1.20
BV82_0797	*phhA*	Phenylalanine-4-hydroxylase	4.82	1.62
*Tryptophan and methionine synthesis*				
BV82_2325	*trpA*	Tryptophan synthase, alpha subunit	− 4.21	− 0.09
BV82_2326	*trpB*	Tryptophan synthase, beta subunit	− 4.28	0.94
BV82_4439	*metE*	5-Methyltetrahydropteroyltriglutamate– homocysteine S-methyltransferase	4.73	0.84
*Efflux pumps of the RND family*				
BV82_0511	*czcA*	acrB/AcrD/AcrF family protein	1.64	− 0.18
BV82_1337		Efflux transporter, RND family, MFP subunit	− 0.38	1.55
BV82_1618		Efflux transporter, RND family, MFP subunit	− 1.91	1.46
BV82_2032	*mexE*	Efflux transporter, RND family, MFP subunit	− 0.18	4.12
BV82_3631	*mdtA_1*	Efflux transporter, RND family, MFP subunit	1.14	− 0.50
*Cell motility and chemotaxis*				
BV82_0871	*fliS*	Flagellar protein FliS	0.36	1.87
BV82_2809		Hypothetical protein/Tfp pilus assembly protein FimV	1.22	4.20
BV82_3217		Methyl-accepting chemotaxis (MCP) signalling domain protein	− 1.82	0.22
BV82_3459	*nirY*	Bacterial regulatory helix-turn-helix, *lysR* family protein Negatively regulates the transcription of the flagellar master operon *flhDC* by binding to the upstream region of the operon	2.22	0.31
BV82_3844		Methyl-accepting chemotaxis (MCP) signalling domain protein	− 2.26	1.22
*Copper tolerance*				
BV82_2904	*copA*	Copper-translocating P-type ATPase	1.50	3.69
BV82_2906	*copZ*	Heavy-metal-associated domain protein	1.97	3.94
BV82_2907	*bcr*	Drug resistance transporter, Bcr/CflA subfamily protein	1.52	3.55

^A^Change in expression upon addition of exudates of a given plant species.

*f.p.* family protein.

The results show that both types of exudates decrease the expression of multiple genes related to the sulfur acquisition. Downregulation of *ssu* genes, or both the *ssu* and *tau* genes, was also observed for 6 out of 8 *Pseudomonas* spp. strains exposed to the root exudates of grass *Brachypodium distachyon*^31^. Maize and tomato plants for the P482 experiments and *B. distachyon* for the experiments with other pseudomonads were grown in Hoagland's medium containing sulfates (MgSO_4_, ZnSO_4_, CuSO_4_). This could potentially influence sulfur availability in the root exudates of the co-cultivated plants. On the other hand, in both studies, cysteine, and methionine were detected in the exudates, which can be a source of organic sulfur for *Pseudomonas* bacteria.

#### Arsenic resistance

The common response of P482 to exudates included upregulation of *arsC* and *arsH* (Table 1). Both genes are related to the metabolism of arsenic, a highly toxic metalloid. Arsenic is ubiquitous in the environment, including soil; therefore, bacteria have developed different mechanisms to process it^32^. ArsC is an arsenate reductase transforming pentavalent arsenate (As(V)) to trivalent arsenite (As(III)) before the efflux of As(III) from cell^33^. The second gene upregulated in P482, *arsH*, confers resistance of certain microorganisms to organoarsenicals like the trivalent forms of herbicide monosodium methylarsenate and the aromatic arsenical phenylarsenite^33^.

It is considered that most methylated arsenic species in the environment are of microbial origin. Some microorganisms were reported to use methylated arsenicals as weapons against their microbial competitors^34^. Plants, unlike microbes, do not methylate arsenicals^35^. Instead, they uptake inorganic arsenicals from the soil in the form of arsenate, likely via the phosphate transporters due to the similarity of those molecules, and they extrude it back to the soil via the roots once it is reduced to trivalent arsenite^36^. We hypothesize that the upregulated expression of *arsC* and *arsH* in P482 could have been triggered by arsenate and organoarsenicals transferred on the roots from the sandy substrate used for the growth of tomato and maize.

#### Iron acquisition from xenosiderophores, heme and intercellular iron homeostasis

One of the COG categories enriched within the shared response was 'Inorganic ion transport and metabolism / Signal transduction mechanism'. This double function category was represented by seven *fecR*-like genes (Table 1). The FecR proteins are transmembrane sensors involved in signal transduction. In *Escherichia coli*, FecR cooperates with the sigma factor FecI and the receptor protein FecA to direct the expression of the *fecABCDE* operon involved in the uptake of ferric citrate^37^. However, bacterial species can harbor many *fecR*-like genes. In the genome of *P. aeruginosa*, there are fourteen *fecR*-like genes located adjacent to iron-starvation sigma factors that can potentially be induced in the presence of different cognate iron carriers^38^. Apart from using self-made siderophores, pseudomonads can use those produced by other bacteria or plants, shifting the energetic cost of their production to other organisms^39^, a phenomenon referred to as 'siderophore piracy'^40^.

Other sources of iron that pseudomonads can use are heme molecules released from hemoproteins^41^. Among the SGRs of P482, the gene showing the most significant downregulation encoded a heme-binding A family protein (− 6.8 log_2_FC) (Table S6). Other genes involved in the uptake and metabolism of heme were also downregulated. These included: *hasR*, *hemO*, *hmuU* and *hmuV,* and *hasD* (Table 1).

To further address how the addition of root exudates influenced iron availability to P482 in our experiments, we have investigated the expression levels of 3 genes: BV82_1009, an NRPS-type synthase involved in the synthesis of the potent siderophore pyoverdine; BV82_1008, (*pvdS*), a sigma factor involved in the regulation of synthesis of pyoverdine, and BV82_4709 involved in the synthesis of 7-hydroxytropolone, a compound with iron-scavenging and antimicrobial properties more characteristic to *P. donghuensis*^11^. Root exudates affected the expression of neither of these genes (Dataset S5). The expression of pyoverdine was also not upregulated in any of the eight *Pseudomonas* strains grown in root exudates of *B. distachyon*^31^. In the latter study, the upregulation of other genes related to iron acquisition was observed only for half of these strains. These included: iron dicitrate transporters (strains SBW25 and 30–84), biosynthesis of siderophores ornicorrugatin (SBW25) and pyochelin (strain Pf-5), heme-degrading enzymes (2–79, 30–84), and TonB-associated genes (2–79 and Pf-5). In another study, the expression of genes related to heme acquisition and pyoverdine synthesis in *P. protegens* CHA0 was relatively low in response to wheat when compared to that observed during the infection of insects^42^.

In contrast to the downregulation of genes for iron acquisition, root exudates of both tomato and maize significantly upregulated the expression of the bacterioferritin gene *bfr* involved in intracellular iron homeostasis. Bfr functions in the storage of iron which, when present in excess in a free form, can cause oxidative stress in the cells through the generation of reactive oxygen species^43^. Bacterioferritin and Bfr-interacting proteins were shown to be essential for stress resistance and virulence in both plant and animal pathogens^44,45^. They also play a role in the root-nodulating plant symbionts^46^.

Upregulation of Bfr in P482 is another indirect premise that root exudates alone are not iron limiting for P482 and at least some other pseudomonads. However, it is highly probable that P482 would need to employ its iron-scavenging mechanisms in the presence of microbial competitors.

#### Assembly of T6SS

Upon exposure of P482 to both exudates, we observed higher expression of *tagQ, pppA*, and *crp*. In *P. aeruginosa*, *tagQ* and *pppA* are involved in the regulatory cascade that controls the activation of the type VI secretion system (T6SS), with *pppA* considered a negative regulator of T6SS^47^. The third gene, *crp*, encodes a cyclic AMP receptor-like protein. The reports on the role of Crp in the regulation of T6SS are scarce and concern *Vibrio cholerae* where Crp was suggested to be a positive T6SS regulator^48^. Overall, our results suggest that the presence of root exudates has some impact on T6SS in P482 grown in monocultures. However, the direction of this impact requires further studies. We can also assume that the expression of T6SS-related genes would be different in the presence of competing (micro)organisms. The T6SS enables bacteria to inject proteins into other procaryotic or eucaryotic cells and is best known for its role in interbacterial competition^49^.

### Tomato-specific response

#### Nitric oxide detoxification and the repair of iron-sulfur clusters

Tomato-treated P482 showed the symptoms of stress caused by nitric oxide (otherwise referred to as nitrosative stress). Nitric oxide is a freely diffusible molecule that exerts toxic effects on cells. To counteract nitrosative stress, bacteria have evolved several pathways to convert NO to non-harmful molecules like N_2_O, NO_3_^−^ or ammonia^50^. One of the two genes with the most substantial upregulation in P482 was the *hmp* (BV82_4743) encoding flavohemoglobin (Hmp). The expression of this gene was highly increased (6.13 log_2_FC) in P482 grown in tomato exudates but not maize. Under aerobic conditions, Hmp catalyzes the reaction of NO with oxygen to yield nitrate (NO_3_^−^)^51^. A similar pattern of changes in gene expression was observed in *norD*, *norC* and *norB* encoding nitric oxide reductase (NOR). This membrane-integrated enzyme catalyzes the reduction of nitric oxide (NO) to nitrous oxide (N_2_O)^52^. Hmp and NOR are well-documented components of the NO-detoxifying mechanism in bacteria.

NO acting on a bacterial cell can originate from the environment or be generated by the cells themselves. NO is a known intermediate in a stepwise reduction of nitrate, through nitrite, to nitrogen oxide^53^. This process, called denitrification, can be performed by some *Pseudomonas* bacteria under limited oxygen availability to overcome the shortage of oxygen as the terminal acceptor of electrons in respiration^54^. The formation of NO from nitrite in the second step of denitrification requires the activity of NirS, a periplasmic cytochrome cd_1_ nitrite reductase carrying heme c and heme d_1_ cofactors^55^. *P. donghuensis* P482 possesses a homolog of *nirS* (BV82_3250) and homologs of multiple other genes of *nir* cluster (*nirCFLGHNBDJM*). In P482 treated with tomato exudates, the upregulation of NO-detoxifying genes was not accompanied by the induction of *nir* genes. Therefore, the underlying cause for the activation of NO-detoxification pathways in P482 is not a switch denitrification but rather coping with the toxicity of exogenous NO present in tomato exudates.

Deleterious effects of NO result predominantly from the inactivation of proteins containing iron-sulfur (Fe-S) clusters^56,57^. Fe-S clusters are redox-active protein cofactors present in almost all organisms and required for many fundamental biochemical processes^58^. In tomato-treated P482, we saw a prominent upregulation of *yftE* (6.36 log_2_FC), known for its role in the metabolism/repair of Fe-S clusters. Homologs of *ytfE* are present in numerous bacteria^59^, and the gene is consistently upregulated upon bacterial exposure to NO^60^. YftE protein was shown to contribute to the survival of *Yersinia pseudotuberculosis* in the spleen following nitrosative stress and contribute to the virulence of this human pathogen^57^. Another gene, the expression of which was significantly induced by tomato exudates, is the *nnrS* (BV82_3241). NnrS is a heme- and copper-containing transmembrane protein that contributes to NO stress tolerance in *Vibrio cholerae* by relieving stress caused by the formation of iron-NO complexes^61^. Noteworthy, the *yftE* and *nnrS* form a single gene cluster (BV82_3238 to BV82_3246) with the NOR-coding genes and two regulators: *norR* and *dnr*. The strain on the Fe-S clusters caused in P482 by tomato exudates is further confirmed by the upregulation of genes *iscR*, *iscS*, *iscU*, *iscA*, *hscB*, *hscA,* and *fdx* (Fig. 3b) encoding proteins related to sulfur cluster biogenesis^62^.

Eucaryotic hosts can produce nitric oxide as a part of defense during infection^63^. Plants use NO as a signal molecule in response to biotic and abiotic stress and a wide range of physiological processes, including germination, development, flowering, and senescence^64^. Since NO is highly reactive, it is stored in S-nitrosothiols (SNOs), which act as NO reservoirs in vivo^64^. NO plays a role in the response of plants to microbial pathogens but is also crucial for establishing symbiotic interactions between rhizobia and legumes^65^. It was suggested that NO might be an essential signaling molecule in communicating between plants and microbes^64^. In the case of P482, it is important to note that the apparent presence of factors causing nitrosative stress in tomato exudates did not negatively influence the growth rate of P482 in vitro (Fig. S1). This implies that P482 is well-adapted to coping with the encountered conditions.

#### Terminal oxidases and respiration

Pseudomonads possess a branched respiratory chain. Five different terminal oxidases are known to operate in *P. aeruginosa*, allowing the bacterium to exploit an electron transport chain best suited under given circumstances^54^. In P482, in response to tomato exudates but not maize, we observed downregulation of *ccoN*, *ccoO*, *ccoQ* and *ccoP* genes encoding the subunits of a *cbb3*-type cytochrome c oxidase. This oxidase has a high affinity for oxygen and is vital for cell survival under microaerobic conditions^66^. Contrary to *cco* genes, *cioA* and *cioB* encoding cyanide-insensitive oxidase (CIO) of the cytochrome *bd* were upregulated. Microorganisms that can switch to the CIO pathway can withstand high concentrations of hydrogen cyanide, which blocks respiration through cytochrome c oxidases^67^. Strain P482 is capable of hydrogen cyanide production^68^. However, in P482 exposed to tomato exudates, genes *hcnA* and *hcnB* encoding the hydrogen cyanide synthase were downregulated, implying that upregulated expression of cyanide-insensitive cytochrome *bd* in this strain does not occur to counter the toxicity of high levels of self-produced hydrogen cyanide.

Although the *cio* genes were initially associated with bacterial resistance to hydrogen cyanide, other factors can affect their expression. Cyanide-insensitive cytochrome *bd* plays a role during copper limitation under aerobic conditions^69^ and confers bacterial tolerance to oxidative and nitrosative stress^70^. Moreover, *cioA* was involved in evading host defenses by *Pseudomonas* sp. WCS365 during interaction with the *Arabidopsis thaliana*^71^.

We hypothesize that a shift towards cytochrome *bd* (CIO) in P482 treated with tomato exudates is due to the presence of inhibitors of cytochrome complex *bc*_*1*_ and/or nitric oxide. Cytochrome complex *bc*_*1*_ is required to pass the electrons to the cytochrome c oxidases like *cbb*_*3*_, but not to CIO which receives electrons directly from ubiquinone^54,72^. The bactericidal effect of *bc*_*1*_ inhibitors was greatly enhanced by the simultaneous addition of NO donors in *Mycobacterium tuberculosis*, an effect bacteria tried to counteract by switching to the cytochrome *bd* pathway^73^. Cytochrome *bd* oxidase is more resistant to NO inhibition due to the fast dissociation rate of NO from its active site.

#### Catabolism of amino acids and the glyoxylate shunt

The intermediary metabolism in P482 was affected predominantly by the exudates of tomato. Three overlapping KEGG pathways were enriched within differentiating response: 'Pyruvate metabolism', 'Propanoate metabolism', and 'Valine, leucine and isoleucine degradation'. Those share genes with enriched COGs related to 'Energy production and conversion' (Fig. 2c, d).

Tomato exudates upregulated the expression of *lpdV*, *bkdB*, *bkdA2,* and *pdhA* (Table 1). Protein products of these genes are the components of the branched-chain alpha-keto dehydrogenase complex, one of three multienzyme complexes metabolizing pyruvate, 2-oxoglutarate, and branched-chain 2-oxo acids. The overall function of these complexes is the conversion of alpha-keto acids to acyl-CoA and CO_2_. Alpha-keto acids often arise by oxidative deamination of amino acids, and reciprocally, they are the precursors for synthesizing thereof^74^. The 2-oxoisovalerate dehydrogenase encoded by *bkdA2* and *pdhA* is an enzyme participating in the degradation of branched-chain amino acids (BCAA): valine, leucine, and isoleucine^75^. In P482, we observed simultaneous upregulation of *mmsA* and *mmsB*. In *P. aeruginosa*, the *mmsAB* operon is involved in the metabolism of valine, and disruption of these genes led to prolonged growth of the strain on the valine/isoleucine medium^76^. In some bacteria, the availability of BCAA appears to have regulatory functions and plays a role in processes such as proliferation during infection and evasion of host defenses^78^. In contrast to what we observed for P482 in the presence of tomato exudates, both *S. typhimurium* and *E. fredii* upregulated synthesis of BCAA, and not their catabolism, when exposed to the exudates of lettuce and intercropped maize, respectively^79,80^.

Apart from BCAA, the catabolism of three other amino acids appears to be induced in P482 upon tomato exudate treatment. This included phenylalanine (genes *phhA*, *phhB,* and *phhC*), glycine (*gcvH* and *gcvP*), and methionine (*aceE/mdeB*, *mdeA*) (Table 1).

Other metabolic changes in P482 associated with increased catabolism of amino acids and/or fatty acids include upregulation of acyl-CoA dehydrogenases MmgC and Ivd, catalyzing the α,β-dehydrogenation of acyl-CoA esters^81^, and the upregulation of *aceA* encoding isocitrate lyase, the latter implying the activation of glyoxylate shunt (GS). Many bacteria activate GS when acetyl-CoA, derived from the catabolism of amino acids or fatty acids, is the primary source of carbon and energy available to the cell^82^. The classical tricarboxylic acid cycle (TCA) cannot assimilate carbon and recycle citrate when acetyl-CoA is the only available carbon source due to the loss of CO_2_ and the inability to recycle oxaloacetate. The GS bypasses the carbon dioxide-producing steps of TCA and diverts part of the carbon flux at isocitrate. Apart from its essential role in acetate and fatty acid metabolism, the glyoxylate shunt may play a still poorly explored role in stress defense and pathogenesis. The expression of *aceA* in *P. aeruginosa* was upregulated under H_2_O_2_-induced oxidative stress and under iron-limiting conditions and is linked to intercellular iron homeostasis^72^. This metabolic pathway is also central for the growth and virulence of *P. aeruginosa* during infection when the bacterium shows a preference for the catabolism of fatty acids^83^. Moreover, shunting of the TCA and the shift toward utilizing amino acids was shown to increase antibiotic resistance in *P. aeruginosa*^84^. Interestingly, the GS was activated in the human pathogen *Salmonella* when grown with lettuce exudates^79^.

Here, we analyzed the initial exudate content (detailed description in SI). LC-SRM analysis of the relative content of amino acids in the exudates of maize and tomato showed that BCAA valine and leucine were approximately 4 times more abundant in maize exudates than in tomato, the content of isoleucine was equal in both samples (Table S7). The quantity of phenylalanine and tryptophan was 4.5 and 2.3 times higher in maize than in tomato, respectively. The abundance of methionine in the two exudates did not differ. Therefore, it is not the case that amino acids, the catabolism of which is upregulated in P482 treated with tomato exudates, are more plentiful in this treatment. In our experimental setup, the influence of exudate supplementation on P482 was assessed in a minimal medium containing glucose. This made glucose the most abundant carbon source in treatments and control. A similar approach was applied in the study on the influence of *B. distachyon* exudates on different *Pseudomonas* spp.^31^. It is possible that P482, in cultures with tomato exudates, exhausted glucose faster than in the maize-supplemented samples. Therefore, at the point of harvest, tomato-treated cells would already use amino acids to support their growth. Non-targeted analysis using ^1^H-NMR revealed that glucose, while highly abundant in maize exudates (9947 ng mg^−1^), was not present in the exudates of tomato (Table S8, Figs. S6, S7). However, it remains questionable if the amount of glucose added with the exudates would be enough to have such an impact on total glucose availability in 1C medium.

We propose that a switch to amino acid catabolism in tomato-treated P482 aims at increasing the resistance of cells to stressors in a similar way it was reported for *P. aeruginosa* in human infection models^83,84^. The presence of NO, causing damage to Fe-S clusters in the active centers of some enzymes, may compromise certain metabolic pathways. In such a case, the observed shift in the intermediary metabolism of P482 would not be driven by a preference for specific carbon sources and their availability in the exudates but rather by choice of metabolic pathways less sensitive to the particular stressor. Plant-derived stressors could be either species-specific or related to a given plant's physiological status. The accuracy of this hypothesis requires further studies.

#### Amino acid synthesis: tryptophan and methionine

In the presence of tomato exudates, the expression of genes encoding tryptophan synthase, *trpA,* and *trpB*, was significantly downregulated, implying reduced synthesis of tryptophan by P482. Tryptophan is a proteinogenic aromatic amino acid that can also be a precursor of signaling molecules such as the plant hormone indole-3-acetic acid (IAA), kynurenine, and the *Pseudomonas quinolone signal* (PQS)^85^. Contrary, we observed significant upregulation of *metE* (4.73 log_2_FC). MetE is a 5-methyltetrahydropteroyltriglutamate, a homocysteine S-methyltransferase that catalyzes the transfer of a methyl group from 5- methyltetrahydrofolate to homocysteine resulting in the formation of methionine. Genes related to methionine synthesis were upregulated in *E. coli* following treatment with S-nitrosoglutathione in conditions mimicking nitrosative stress. In the latter study, *E. coli* mutants disrupted in the methionine synthesis pathway exhibited decreased tolerance to S-nitrosoglutathione and that the addition of exogenous methionine could abrogate this effect^86^. This suggests that methionine could also play a role in counteracting nitrosative stress in P482.

Moreover, methionine synthesis was essential for establishing symbiosis between nitrogen-fixing *Sinorhizobium meliloti* and its plant host, *Medicago sativa*^87^. Contrary to what we observed in P482 in the presence of root exudates of tomato, methionine synthesis was decreased in PAO1 in response to exudates of beetroot^25^ and in *S. typhimurium* in response to the exudates of lettuce^79^. This suggests that the role of methionine synthesis in plant-associated bacteria is dependent on the studied plant–microbe system.

In the context of how amino acid synthesis and catabolism are affected in tomato-treated P482, it is worth mentioning that these may also have less straightforward causes. In *P. fluorescens,* the phenylalanine 4-hydroxylase (PAH) is involved not only in the catabolism of phenylalanine but also in the biosynthesis of antioxidant melatonin from tryptophan^88,89^. It was also shown that tryptophan, methionine, tyrosine, and phenylalanine, the well-known precursors of plant secondary metabolites, may contribute to increased plant resistance^90^. It can be speculated that P482, by degrading phenylalanine, reducing the synthesis of tryptophan, and balancing the levels of methionine, may try to counteract and influence what "it interprets" as the immune response of the plant.

### Maize-specific response

#### MexE efflux pump of the RND family

Maize exudates caused the upregulation of three Membrane Fusion Protein (MFP) subunits of RND exporters: *mexE*, BV82_1337, and BV82_1618. The expression of *mexE* was particularly upregulated (4.12 log_2_FC). RND stands for Resistance-Nodulation-Division superfamily of efflux pumps. Efflux pumps of the RND superfamily and the Omp proteins form protein complexes that enable Gram-negative bacteria to export harmful drugs directly outside the cell and not, like in the case of other efflux systems, into the periplasmatic space. This makes RND efflux pumps important determinants of multidrug resistance^91^. In *P. aeruginosa*, several efflux operons confer intrinsic resistance of the bacterium to antibiotics, with *mexE*-containing operon *mexEF-oprN* conferring resistance to quinolones, chloramphenicol, and trimethoprim^92^. However, it is likely that the resistance to synthetic antibiotics is just a side-effect of resistance to natural secondary metabolites. The expression of *smeDEF* was shown to be triggered by plant-produced flavonoids, and that the inactivation of the pump by deletion of *smeE* impaired the ability of *Stenotrophomona maltophilia* to colonize the roots of the oilseed rape plant^93^. It is, therefore, plausible that the overexpression of *mexE* in P482 in response to the exudates of maize is involved in counteracting the presence of particular secondary metabolites specific to this plant host. In line with the host-specific effect is that only particular, and not all the RND transporters were induced in the presence of maize. The upregulation of *czcA* and *mdtA,* also encoding RND efflux pumps, was driven by tomato (Table 1). Maize and other grasses (*Poaceae*) are known to produce and secrete biocidal benzoxazinoids, compounds with an established role in shaping the plant microbiome^27,94^. It appears worth investigating if RND transporters play a role in the tolerance of some bacteria to benzoxazinoids.

#### Copper tolerance

Both exudates upregulated the expression of *copA* and *copZ* in P482*.* However, the induction of those genes was much higher in response to maize (Table 1). CopA is a transmembrane ATPase responsible for the efflux of Cu^+^ ions delivered by the cytoplasmic chaperone CopZ. Both proteins are elements of well-characterized Cu^+^ tolerance systems^95^. Copper is an essential trace element for all organisms; however, it is toxic in excess^96^. Copper in the form of CuSO_4_ is an ingredient of Hoagland's plant growth medium, used in this study to culture both tomato and maize. Therefore, the fact that copper tolerance response is so much more robust in maize exudates than in tomato needs to be caused by host-dependent factors affecting either the availability or toxicity of copper. Mavrodi and co-workers^31^ showed considerable upregulation of one or more *cop* genes related to copper tolerance in 5 out of 8 investigated pseudomonads treated with the exudates of *B. distachyon*. Both maize and *B. distachyon* are Monocot grasses, and both were cultured in Hoagland's for the respective experiments.

### Cell motility is upregulated by maize exudates but downregulated by tomato

In P482, maize exudates caused upregulation of BV82_2809 encoding FimV, a protein involved in the assembly of type IV pili. The type IV pili are involved in adherence, certain types of movement (a social gliding in *Myxococcus xanthus* and twitching in *Pseudomonas* and *Neisseria* species), and biofilm formation, guided tissue invasion, and other pathogenesis-related events^97^. Type IV pili are essential for endophytic rice colonization by the N2-fixing endophyte *Azoarcus* sp. strain BH72^98^. Interestingly, *pilK*, involved in twitching motility, was downregulated in *P. aeruginosa* in response to the exudates of beetroot^25^, suggesting that beetroot exudates have a down tuning effect on pili formation in *P. aeruginosa,* as opposed to the stimulative effect of maize compounds in *P. donghuensis*.

Maize exudates caused upregulated expression of *fliS* in P482*,* suggesting increased synthesis of swimming-related flagella. Flagella are essential for some *Pseudomonas* strains to colonize their hosts efficiently. *P. fluorescens* F113 with a disrupted *fliS* gene was non-motile and unable to compete with the wild type (WT) strain for colonizing the root tip of alfalfa^99^.

Unlike in maize-treated cells, upregulation of genes related to the synthesis of pili and flagella was not observed in P482 treated with tomato exudates. Quite the opposite, tomato exudates upregulated the expression of a presumed negative regulator of the flagellar master operon *flhDC* in locus BV82_3459. It was shown that although flagella can provide an advantage to the cells, their synthesis may also lead to some disadvantages. Recognition of specific flagellar antigens may induce a hypersensitive response and cell death as part of the host's immune response^100^. A decrease in flagella synthesis is considered a major mechanism in evading plant immunity^101^. Studies on *P. putida* KT2440 concerning the metabolic trade-offs of producing flagella showed that a non-flagellated mutant strain presents a shorter lag phase and is more resistant to oxidative stresses than the WT strain. It was suggested that lack of the metabolic expense on producing flagella might provide cells with a surplus of energy (ATP) and reduce power (NADPH) that the cells can allocate for other activities, including stress resistance^102^. It, therefore, seems that the advantages of having lower expression of flagellar genes outweigh the potential fitness disadvantages in P482 treated with tomato exudates. Tomato exudates also downregulated two genes encoding methyl-accepting chemotaxis signaling domain proteins (MCPs), which function as the predominant chemoreceptors in bacteria and archaea^103^.

Taken together, maize exudates increased the expression of motility-related functions in P482, while the opposite was observed in response to the exudates of tomato. It remains to be elucidated if those differences are related to the varying importance of motility in the colonization of these two hosts, or rather it was the physiological state of the tomato sampled for exudates in our study which made the expression of those genes unfavorable.

### Conclusions and outlook

**Figure 4:**
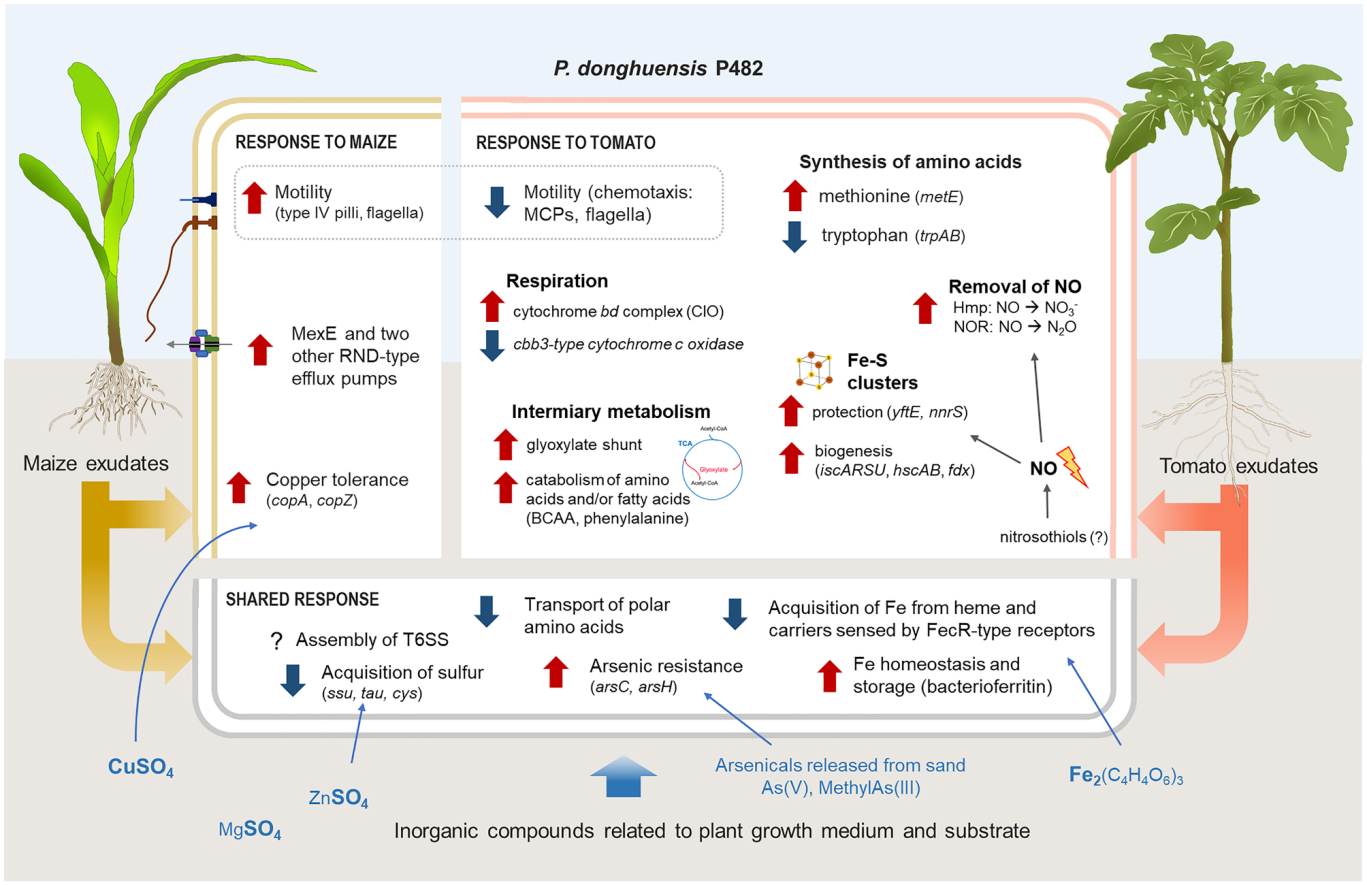
Shared and host-specific transcriptomic responses of *P. donghuensis* P482 to the exudates of tomato and maize. Red arrows pointing upward indicate the upregulation of a pathway, while blue arrows pointing downward indicate downregulation.

Understanding plant–microbe interactions is a key requirement for harnessing the potential of microbes to support plant health. Studying those interactions in the full complexity of natural systems remains technically challenging. Therefore, simplified setups that isolate selected interactions continue to help gain valuable insights^31,104^. Here, we identified which aspects of the metabolism of *P. donghuensis* P482 are differently affected by root exudates of two different plant hosts: tomato and maize (Fig. 4). The composition of tested exudates, and therefore their influence on P482, are most likely the result of both the genotype of the sampled plants and their physiological status. We conclude that catabolism of branched-chain amino acids switches to glyoxylate shunt, resistance to nitric oxide, flexible use of respiratory chain, methionine synthesis, and selective activation of RND-type efflux pumps deserve particular attention in terms of their role in how promiscuous root colonizing *Pseudomonads* adapt to different plant hosts, and how they stay associated when challenged with a change in the host's physiological status.

Much attention concerning microbiome assembly is devoted to the attraction between the microbes and the plant. Contradictory to this trend, most of the host-specific pathways we have described in P482 are related to stress resistance. Despite that, the exudates stimulated and did not inhibit the growth of P482. Higher resilience of some microbes to particular stressors may lead to shifts in plant-associated microbial populations, particularly in plants with activated stress responses. In parallel to human social behavior, not being bothered by one's partner's outbursts may be an underrated factor in whether one decides to stick around.

## Supplementary Information

Supplementary Information 1.

Supplementary Information 2.

Supplementary Information 3.

Supplementary Information 4.

Supplementary Information 5.

Supplementary Information 6.

Supplementary Information 7.

## Data Availability

Raw sequencing reads for this study are available in the NCBI Sequencing Read Database (PRJNA868728). Differential gene analysis files were deposited in NCBI Gene Expression Omnibus (GSE211040).
