# Supplementary material for: Host-adaptive traits in the plant-colonizing Pseudomonas donghuensis P482 revealed by transcriptomic responses to exudates of tomato and maize
Source: Sci Rep. 2023 Jun 9;13:9445. doi: 10.1038/s41598-023-36494-6 (PMC10256816; doi:10.1038/s41598-023-36494-6)
Supplement: Supplementary file 7 — Supplementary Information 7. [file 41598_2023_36494_MOESM7_ESM.pdf]

Supplementary Information

Host-adaptive traits in the plant-colonizing *Pseudomonas donghuensis* P482 revealed by transcriptomic responses to exudates of tomato and maize

Dorota M. Krzyżanowska[1], Magdalena Jabłońska[1], Zbigniew Kaczyński[2], Małgorzata Czerwicka-Pach[2], Katarzyna Macur[3], Sylwia Jafra[1,*]

*** Correspondence:** Sylwia Jafra: sylwia.jafra@ug.edu.pl

1 Supplementary Materials and Methods

1.1 Bacterial strains and culture conditions

Bacterial strains used in this study included *Pseudomonas donghuensis* P482, isolated from the rhizosphere of tomato [1,2], and its spontaneous rifampicin-resistant mutant P482 Rif [3]. Routine propagation of bacteria was performed at 28 °C in Miller's LB liquid medium (Roth), with agitation (120 rpm), or on solid media plates with LB or TSB (Oxoid) supplemented with agar (15 g L^{-1}). Where indicated, rifampicin (50 µg mL^{-1}) (Merck), ampicillin (50 µg mL^{-1}) (Polfa Tarchonin), and cycloheximide (100 µg mL^{-1}) (Merck) were supplemented to the media as selective agents.

1.2 Colonization of roots of plants grown in soil

Tomato seeds were sterilized by treating with 70% ethanol for 1 min, followed by 3 min in 3% NaOCl and 3 times rinsing in sterile distilled water. Seeds of maize were surface sterilized by treating twice with 3% NaOCl for 15 min, followed by 10 min in 70% ethanol and 3 times rinsing with sterile distilled water. Seeds were germinated for 4 days in glass Petri dishes lined with Whatman filter paper. The filter paper was moistened with distilled water and the plates were sealed with parafilm to retain moisture. Tomato was germinated in the dark at 24 °C while maize was germinated in the light at 22 °C (optimal conditions for germination depending on the species).

To obtain bacterial inoculum, strain P482 Rif was cultured for 24 h on TSB agar supplemented with rifampicin (50 µg mL^{-1}). Cells were harvested, suspended in sterile saline and the turbidity of the suspension was adjusted to 6 units in McFarland scale (10^9 colony forming units per mL; CFU mL^{-1}). The cells were harvested by centrifugation (4 min, 3800 RCF). The pellet was re-suspended in 1% carboxymethylcellulose (Calbiochem, Germany), in a volume equal to that of the discarded supernatant. The germinated seeds were coated with the inoculant by brief submersion and placed in the soil in 12 cm dia. pots. Tomato was grown in universal potting soil (Kronen) and maize was grown in a mixture of potting soil and silica sand (1:1, v/v) to assure proper drainage of the substrate. Seven pots were prepared per plant species, with one seed per pot for maize and three seeds per pot in the case of tomato. Plants were grown for 14 days in a phytotron chamber, 16/8 h

day/night photoperiod, 22 °C, 60% humidity, white light (Philips Master TL-D 36W/840 5B, Philips).

After 14 days of growth in soil (18 days in total, including germination), plants were removed from the pots. Roots were shaken to remove any unbound soil and were placed in universal filter extraction bags (Bioreba). Nearly complete root systems were collected to avoid any biases that might result from variations in the special distribution of bacterial cells on the root. Nine mL of sterile saline was added to each extraction bag per 1 g of sample. The bag was sealed, and plant material was homogenized. Immediately after homogenization, each suspension was serially diluted and 10 μL aliquots of each dilution were spotted onto TSB agar supplemented with rifampicin (50 μg mL^{-1}) and ampicillin (50 μg mL^{-1}), and cycloheximide (100 μg mL^{-1}), with three technical replicates each, as described previously [4]. Plates were incubated at 28 °C for up to 48 hours and colonies enumerated. The titer of P482 Rif was normalized for the mass of the rhizosphere samples. The experiment was performed twice.

Data from the duplicated experiments were pooled for statistical analysis to obtain 14 data points per species. The normality of data was determined using Shapiro-Wilk's test. Non-parametric two-tailed Mann–Whitney U test (α=0.05) was applied to determine the significance of differences between groups, with the help of an online tool available at http://www.statskingdom.com/.

1.3 Chemical analysis of the composition of root exudates

1.3.1 GC-MS

Samples for GC-MS were prepared as described in [5]. Twenty mL aliquots of water-collected exudates of tomato and maize were acidified to pH 2 with 0.5 M HCl, freeze dried and silylated in 60 μL of 1:1 (v:v) acetonitrile:N-tert-Butyldimethylsilyl-N-methyltrifluoroacetamide (MTBSTFA, Acros Organics). Derivatization was performed by sonication in glass chromatographic bottles at 60 °C for 3 h in an ultrasonic bath, followed by an overnight incubation at room temperature. GC-MS analysis was performed with GCMS-QP2010 SE (Shimadzu). One μL of each sample was separated on a capillary column DB-5MS (30 m × 0.25 mm i.d.) coated with 0.25 μm film (5%-phenyl)-methylpolysiloxane, bonded and cross-linked, with helium gas as carrier. The temperature of the column was set to 60 °C for 2 min, followed by 20 °C min^{-1} increment to 150 °C, and then 6 °C min^{-1} to 290 °C. The temperature of injection (splitless), transfer line and ion source were set to 260 °C, 290 °C and 200 °C, respectively. The MS was operated in full-scan with range from 45 to 650 m/z and EI ionization (70 eV). Data analysis was performed in Lab Solutions with NIST 11 Mass Spectral Library.

Relative quantitative analysis was performed based on a four-point standard curve for lactic acid (0.00001-0.01 mg mL^{-1}, R^2=0.999) (Acros Organics) and, to establish compound-specific response of the detector, two mixed compound standards, each comprising lactic acid and either nine or five other compounds (Table S10). All standards were processed and analyzed by GC-MS as described for the samples.

1.3.2 NMR

For each of the two plant species, two hundred mL of water-collected exudates were freeze dried. The lyophilized compounds were suspended in 20 mL of sterile high-purity water and treated with 1 g of sterile Chelex 100 resin (Bio-Rad), in a batch extraction, to remove NMR-interfering polyvalent metal ions. The resin was removed by centrifugation (5 min, 8500 RCF), and the pH of the samples was adjusted to ~pH 7 with 0.5 M HCl. The aliquots were freeze dried again and the dry weight of the plant-derived compounds was established. Each sample was re-suspended in 0.65 mL of D_2O and ^1H NMR spectra were obtained with Bruker AVANCE III 500 MHz (Bruker).

1.3.3 Liquid Chromatography-Selected Reaction Monitoring (LC-SRM)

1.3.3.1 Sample preparation

The stock solutions of 21 standard L-amino acids (1mg/mL): alanine (Ala, Acros), aspartic acid (Asp, MP Biomedicals), cysteine (Cys, BioShop), leucine (Leu, Acros), tyrosine (Tyr, Acros), phenylalanine (Phe, Acros), ornithine (Orn, Alfa Aesar), arginine (Arg, BioShop), glutamic acid (Glu, MP Biomedicals), glutamine (Gln, Roth), glycine (Gly, Sigma Aldrich), histidine (His, BioShop), lysine (Lys, Acros), methionine (Met, MP Biomedicals), proline (Pro, BioShop), tryptophan (Trp, BioShop), taurine (Tau, Alfa Aesar), serine (Ser, Acros), isoleucine (Ile, SAFC), threonine (Thr, Sigma Aldrich), valine (Val, BioShop) and deuterated leucine (LeuD3, L-Leucine-5,5,5-d3, 99% atom D, Sigma-Aldrich), all of at least ≥ 98.5 purity, were prepared in 0.1 M HCl (Sigma Aldrich) and further diluted with water (LC-MS grade, Merck) before the LC-MS/MS experiments. The aqueous root exudates of maize and tomato samples (100 µL each) were spiked with deuterated leucine standard (Leu D3, Sigma Aldrich, 50 ng/mL in the final sample) and evaporated to dryness using vacuum concentrator. Then, they were dissolved in 10:90 of mobile phase A (10 mM ammonium formate in water) and B (10mM ammonium formate in acetonitrile) (all LC-MS grade, purchased at Merck) and transferred to the LC vials.

1.3.3.2 Measurments

Relative quantification of amino acids in maize and tomato root exudates was performed on QTRAP 6500 triple quadrupole tandem mass spectrometer (MS/MS) (SCIEX) coupled in-line with Eksigent LC200 (Eksigent) microLC system, which were controlled by Analyst 1.6.2 software (SCIEX). Five µL of each of the samples were injected in five technical replicates each by the CTC PAL autosampler onto the Reprospher HILIC-A 3 µm 150 x 0.5 mm column (Dr Maisch), where their components were separated using mobile phase A and B and the following gradient program: 0-0.5 min – 1% A /99 % B, 0.5-12min – 1% A to 30% A, 12-14min – 30% A / 70 % B, 14-16 min – 30% A to 1% A, 16-20 min 1% A /99 % B (with column equilibration at 1% A /99% B for 20 min), at the flow rate of 20 µL/min. The eluates from the column were subsequently ionized in the positive ion mode in the TurboV ESI ion source (SCIEX), which was working at 5500 V ion spray voltage, 30 curtain gas, 30 ion source gas 1 and 2, medium collision gas and 300°C temperature. Finally ionized

samples were subjected to the MS/MS analyses in the multiple reaction monitoring (MRM) mode at de-clustering potential (DP), collision energy (CE) and cell exit potential (CXP) individually optimized for each analyte (Table S11); entrance potential of 10 V, 20 ms dwell time, unit resolution and 5 ms pause between mass ranges. Standard solutions of amino acids and syringe infusion to the mass spectrometer were used for manual development of two MRM transitions for each amino acid (one qualifier, one quantifier) for the LC-MS/MS method and to determine their compounds parameters, namely, DP, CE and CXP (Table S11).

1.3.3.3 Data analysis

The .wiff files from LC-MS/MS analysis were exported to the MultiQuant 2.1 software (SCIEX). The following parameters of the MQ4 algorithm was applied for extraction of areas under peaks of each MRM transition: Gaussian smooth 1.0 point, expected RT defined for each amino acid separately, RT half window 30 sec, report the largest peak, minimum peak width 2-point, minimum peak height 0; integration parameters: noise percentage 40 %, baseline subtraction window 2 min, peak splitting factor 2 points. The extracted areas under peaks of the quantifier MRM transitions for each amino acid (Table S11) were then exported to Excel, where the mean area under peak of 5 technical replicates for each sample was calculated. The $\log_2$ fold change in each of the analyzed amino acids in maize versus tomato samples along with its p-value, determined in two-sided unequal variance t-Test, were then also calculated using features available in Excel.

2 Supplementary Results

2.1 Differences in the composition of root exudates of tomato and maize

The composition of exudates of 18 days old tomato and maize plants was analyzed by GC-MS and ^{1}H-NMR. The obtained chemical profiles were compared to identify major differences between the studied cultivars of the two plant species.

GC-MS analysis revealed 72 picks in total for tomato in comparison to 63 picks for maize (**Fig. S6**). Out of compounds successfully identified and quantified, the most abundant were found to be, in the exudates of tomato: malic acid (88 ng mg^{-1} of exudate dry weight), azelaic acid (72 ng mg^{-1}), succinic acid (53 ng mg^{-1}) and lactic acid (49 ng mg^{-1}) (Table S9) and, in the exudates of maize: malic acid (1481 ng mg^{-1}), citric acid (175 ng mg^{-1}), trans-aconitic acid (135 ng mg^{-1}), lactic acid (93 ng mg^{-1}) leucine (41 ng mg^{-1}) and succinic acid (37 ng mg^{-1}).

A limitation of GC-MS is that, within the sample, only compounds that undergo silylation can be analyzed. On the contrary, ^{1}H-NMR enables unbiased measurement of whole samples, however yielding complex spectra that can pose interpretational challenges. In this study, ^{1}H-NMR analysis of exudate samples confirmed the presence of malic acid and citric acid in both samples, as determined by GC-MS. Moreover, ^{1}H-NMR revealed the presence of high amounts of glucose (9947 ng mg^{-1}) in the exudates of maize – a sugar not detected in the tomato-derived samples (Fig. S7; Table S8). A more general conclusion brought by ^{1}H-NMR analysis was that while tomato exudates contained relatively small amounts of multiple compounds, the composition of exudates of maize was less versatile but the compounds were present in higher concentrations.

Compounds present solely in one type of exudate, or present in significantly higher amounts were considered characteristic for a given plant species. In the case of tomato, this included pimelic acid and azelaic acid. Each mg of dry weight of the exudates of tomato contained 16 times more pimelic acid and 10 times more azelaic acid than was detected in the exudates of maize. Compounds more typical for maize were glucose, aconitic acid (both not detected in tomato), citric acid, leucine, and phenylalanine. Exudates of maize also contained more malic acid than the exudates of tomato. However, as determined by GC-MS, malic acid was the most highly abundant compound in the exudates of both species (Table S8).

Relative quantification of 21 amino acids in the exudates of maize and tomato was performed using HILIC LC-MS/MS in MRM mode (here referred to as LC-SRM). The method was developed and optimized for this purpose. Differences in the quantity of amino acid between the analyzed samples were expressed as $\log_2$ fold changes (Table S7). Fourteen amino acids were more abundant in the exudates of maize compared to the exudates of tomato, out of which differences for 10 were statistically significant ($p<0.05$): alanine, arginine, leucine, lysine, ornithine, phenylalanine, proline, threonine, tryptophan, and valine. In turn, 5 amino acids were less abundant in maize exudates than in tomato exudates, of which the difference was statistically significant solely for taurine (Table S7).

2.1.1 Pathways of the shared response (not mentioned in the main text)

2.1.1.1 Transport of positively charged amino acids

Both exudate treatments downregulated the expression of *aotQ*, *hisM* and BV82_1073, all of which encode ABC transporters. AotQ is an arginine/ornithine transport protein, while HisM is a part of $HisQMP_2$ transporter for positively charged amino acids (lysine, arginine, histidine) [6]. The last gene, BV82_1073, encodes a solute-binding protein, presumably involved in amino acid transport and metabolism and/or signal transduction. Taken together, the addition of both types of exudates led to the downregulation of certain amino acid transport pathways in strain P482.

2.1.1.2 Shikimate pathway

One of the top-ranking downregulated SRGs was the *aroF* (Table S6). The gene encodes 3-deoxy-7-phosphoheptulonate synthase (DAHP), the first enzyme of the shikimate pathway. This pathway is involved in the synthesis of folates (vitamin B_9) and aromatic amino acids: phenylalanine, tyrosine and tryptophan, among other metabolites. This indicates a decreased demand for these compounds in P482 in the presence of exudates.

2.1.2 Pathways of the differentiating response (not mentioned in the main text)

2.1.2.1 Genes of phage origin

In the presence of tomato exudates, but not maize exudates, we observed a significant upregulation of BV82_0566, BV82_0567, BV82_0568 (*gpV*), and BV82_0571. These genes are annotated as encoding a putative holin, a hypothetical protein, a phage baseplate assembly V family protein, and a phage tail protein I, respectively. They are thus likely of phage origin, although they are only remnants of a phage that was once incorporated in the strain. Our recent *in silico* screening of the complete genome of P482 for the presence of prophages did not reveal an intact/complete prophage in this genomic region [7]. It is noteworthy that genes of phage origin are induced in the presence of tomato exudates, supporting the premise that P482 experiences some form of stress in this exudate given that prophage induction is known to occur following treatment with toxic chemicals or UV light [8].

3 Supplementary Tables

Table S1 Statistics after demultiplexing and filtering.

Sample name	Number of reads	Yield in mbp	Average quality
P482_maize_1	30925123	9066	35.7
P482_maize_2	28740810	8431	35.66
P482_maize_3	29182676	8600	35.62
P482_tomato_1	24442543	7283	35.8
P482_tomato_2	27623025	8162	35.63
P482_tomato_3	31350329	9278	35.61
P482_CTRL_1	25404161	7564	35.68
P482_CTRL_2	28791672	8477	35.87
P482_CTRL_3	20810455	5844	35.96

More information about the average quality score can be found at the Illumina website (https://www.illumina.com/science/education/sequencing-quality-scores.html)

Table S2 RNAseq alignment statistics.

Sample name	Reference genome (Genbank)[A]	Filtered reads	Unique reads (%)	Multimapped reads (%)	Unmapped reads (%)	Reads per uniqly mapped (in millions)
P482_maize_1	JHTS00000000.1	30925123	15.9	64.79	19.31	4.92
P482_maize_2	JHTS00000000.1	28740810	17.75	63.31	18.94	5.10
P482_maize_3	JHTS00000000.1	29182676	14.14	64.68	21.18	4.13
P482_tomato_1	JHTS00000000.1	24442543	5.3	72.93	21.77	1.30
P482_tomato_2	JHTS00000000.1	27623025	7.54	71.46	21	2.08
P482_tomato_3	JHTS00000000.1	31350329	9.36	68.9	21.74	2.93
P482_CTRL_1	JHTS00000000.1	25404161	15.74	64.23	20.03	4.00
P482_CTRL_2	JHTS00000000.1	28791672	15.77	65.73	18.5	4.54
P482_CTRL_3	JHTS00000000.1	20810455	19.13	67.56	13.31	3.98

[A] Reference number of sequences (contigs): 69. Reference number of bases: 5717769.

Table S3 List of PCR primers used in this study with the established amplification efficiencies.

Target gene (locus)	Primers	Primer sequences	Amplicon length (bp)	E^A	SE (E)^B	$R^{2\,C}$	Slope	Reference
gyrB (BV82_2296)	F_ gyrB<br>R_ gyrB	5’ ATCGACAAGCTGCGCTATCA<br>5’ CGGCTGAGCGATGTAGATGT	144	1.96	0.08	0.981	-3.41	[9]
rpoD (BV82_1895)	F_ rpoD<br>R_ rpoD	5’ CCACGACGGTATTCGAACTT<br>5’ CGTGCCAAGAAAGAAATGGT	152	1.96	0.02	0.999	-3.42	[9]
lrp (BV82_3254)	F_lrp<br>R_lrp	5’ CCCGAGGTCAACCACAACTA<br>5’ GTGTCGGCTTCCAGTTCGT	113	1.944	0.008	1	-3.463	This study
mexE (BV82_2032)	F_mexE<br>R_mexE	5’ GCGTTACCCCCTTGCTCTAT<br>5’ GTGAACTCGTCCCATTCGTT	141	1.956	0.012	0.999	-3.432	This study
norC (BV82_3246)	F_norC<br>R_norC	5’ ATGCATGCCTGGATGAAGAT<br>5’ TGATGTTCGAGCTCCATTTG	124	1.999	0.013	0.999	-3.325	This study
ssuC (BV82_1676)	F_ssuC<br>R_ssuC	5’ GCACCTTGTTCCCGATTTAC<br>5’ AGAATCACCTGGCGAAACAG	121	1.97	0.017	0.998	-3.396	This study
trpB (BV82_2326)	F_trpB<br>R_trpB	5’ AATCGATCATCGGCAAAGAG<br>5’ ATCGAGGAAGTCGTGGAACA	125	2.114	0.027	0.997	-3.024	This study
ytfE (BV82_3239)	F_ytfE<br>R_ytfE	5’ GACATGCAGCAGGAACTTGA<br>5’ ATGCTCCAGACGCATAACCT	120	1.976	0.015	0.999	-3.381	This study

[A] primer pair efficiency; value established based on 7-point standard curves for which 10-fold serial dilutions of post-PCR products were used as templates.

[B] efficiency standard error

[C] coefficient of determination for linear regression calculated for a 7-point, 10-fold dilution standard curve

Table S4 Tomato-specific genes of the differentiating response (GDRs) with the highest change in expression.

Locus	Gene	Log_2FC	Annotation
Upregulated			
BV82_3239	*ytfE*	6.36	hemerythrin HHE cation binding domain protein
BV82_4743	*hmp*	6.13	oxidoreductase NAD-binding domain protein
BV82_3240		5.54	putative membrane protein
BV82_3241	*nnrS*	4.99	nnrS family protein
BV82_0044	*cioA*	4.92	bacterial Cytochrome Ubiquinol Oxidase family protein
BV82_4439	*metE*	4.73	5-methyltetrahydropteroyltriglutamate-- homocysteine S-methyltransferase
BV82_0046		4.50	hypothetical protein
Downregulated			
BV82_3674		-4.48	ABC transporter, substrate-binding, aliphatic sulfonates family protein
BV82_1669	*tauA*	-4.32	taurine ABC transporter, periplasmic binding protein
BV82_2326	*trpB*	-4.28	tryptophan synthase, beta subunit
BV82_2325	*trpA*	-4.21	tryptophan synthase, alpha subunit

Table S5 Maize-specific GDRs with the highest change in expression.

Locus	Gene	Log_2FC	Annotation
Upregulated			
BV82_3988		4.96	hypothetical protein
BV82_2809		4.20	hypothetical protein
BV82_2032	*mexE*	4.12	efflux transporter. RND family. MFP subunit
BV82_2904	*copA*	3.69	copper-translocating P-type ATPase
BV82_4275	*slyA1*	3.25	marR family protein
BV82_2815	*ripA_1*	3.14	bacterial regulatory helix-turn-helix s. AraC family protein
BV82_1378		2.86	bacterial regulatory s. tetR family protein
BV82_1235	*nuoA*	2.77	NADH-ubiquinone/plastoquinone oxidoreductase. chain 3 family protein
Downregulated			
BV82_3753		-3.12	tonB-dependent siderophore receptor
BV82_1217	*furB*	-2.31	Fe^{2+} Zn^{2+} uptake regulation protein
BV82_1856	*polC1*	-2.04	exonuclease family protein
BV82_1868	*bioF*	-1.96	8-amino-7-oxononanoate synthase
BV82_0119	*phrB*	-1.93	FAD binding domain of DNA photolyase family protein
BV82_2744	*thiC*	-1.90	thiamine biosynthesis protein ThiC
BV82_0367	*gcvH*	-1.63	glycine cleavage system H protein
BV82_3597	*rstB*	-1.51	HAMP domain protein

Table S6 Genes of the shared response to exudates (SGRs) with the highest change in expression.

Locus	Gene	Log_2FC Tomato	Log_2FC Maize	Annotation
Upregulated				
BV82_3873	*arsC*	2.42	2.66	low molecular weight phosphotyrosine phosphatase family protein
BV82_3874	*arsH*	2.63	2.35	arsenical resistance protein ArsH
BV82_2350	*surf1*	3.02	1.84	SURF1 family protein
BV82_5016	*fdnG*	2.70	2.08	formate dehydrogenase. alpha subunit
BV82_4419	*bfr*	1.87	2.67	bacterioferritin
BV82_2984		2.41	1.85	plasmid replication region DNA-binding N-term family protein
BV82_1931	*crp*	2.03	2.05	cyclic AMP receptor-like protein
Downregulated				
BV82_0056		-6.83	-6.84	heme-binding A family protein
BV82_1668	*tauB*	-4.52	-1.87	ABC transporter family protein
BV82_1666	*tauD*	-4.11	-3.83	alpha-ketoglutarate-dependent taurine dioxygenase
BV82_1667	*tauC*	-4.05	-3.65	binding--dependent transport system inner membrane component family protein
BV82_3001		-3.89	-3.35	hypothetical protein
BV82_0058	*hasE*	-3.87	-3.32	type I secretion membrane fusion, HlyD family protein
BV82_0097		-3.66	-4.31	lysE type translocator family protein
BV82_3002	*aroF*	-3.10	-3.35	3-deoxy-7-phosphoheptulonate synthase (DAHP)

Table S7 The calculated $\log_2$ fold differences in the quantity of 21 amino acids in maize root exudates compared to tomato root exudates, with their corresponding p-values of statistical significance.

Amino acid	log2 fold difference[A]	p-value	Statistical significance (p<0.05)
Ala	1.6542	1.14 E-02	yes
Arg	2.5996	2.05 E-06	yes
Asp	0.9521	6.49 E-02	no
Cys	-1.5467	1.90 E-01	no
Gln	-0.2455	7.51 E-01	no
Glu	-1.7185	8.94 E-02	no
Gly	-0.2519	8.29 E-01	no
His	2.3553	5.66 E-02	no
Ile	0.9354	6.30 E-02	no
Leu	2.2029	1.58 E-02	yes
Lys	0.9745	2.14 E-03	yes
Met	0.5612	1.53 E-01	no
Orn	1.4055	5.23 E-07	yes
Phe	2.1967	5.54 E-03	yes
Pro	2.7591	9.17 E-11	yes
Ser	-0.5485	5.61 E-01	no
Tau	-3.1630	3.00 E-10	yes
Thr	1.9070	2.28 E-02	yes
Trp	1.2491	8.15 E-04	yes
Tyr	2.7140	5.05 E-08	yes
Val	2.3742	2.23 E-05	yes

[A] tomato exudates were used as the reference sample.

Table S8 GC-MS and NMR quantification of tomato and maize exudates collected in water.

Compound	Maize (ng/mg)	Tomato (ng/mg)	Ratio (Maize/ Tomato)
Glucose	9947.00	DNQ	-
trans-Aconitic acid	134.56	ND	-
Citric acid	175.40	3.99	43.98
Malic acid	1481.02	88.13	16.80
L-Leucine	40.63	3.21	12.66
L-Phenylalanine	19.65	3.39	5.80
Lactic acid	92.63	48.62	1.91
Glycolic acid	8.80	4.94	1.78
Myristic acid	22.60	17.72	1.28
Palmitic acid	6.28	5.74	1.09
Stearic acid	8.28	8.70	0.95
Succinic acid	36.92	53.42	0.69
Azelaic acid	7.19	71.85	0.10
Pimelic acid	1.06	16.91	0.06
Glycine	DNQ	DNQ	-
L-Tyrosine	DNQ	DNQ	-
Oleamide	DNQ	DNQ	-

The quantity of compounds in given in ng per mg of exudate dry weight. Quantified by GC-MS using a mixture of standards, with the exceptions glucose, the latter quantified with NMR using lactic acid and malic acid as internal standards.

DNQ – detected not quantified (weak signal)

ND – not detected

Table S9 Composition of standards for GC-MS.

Compound	CAS no.	Supplier	Cat. no.
Mix 10			
5-Chloroindole-2-carboxylic acid	CAS 10517-21-2	Alfa Aesar	A18626.03
Azelaic acid	CAS 123-99-9	Acros Organics	401520250
4-Methoxyphenylacetic acid	CAS 104-01-8	Acros Organics	126021000
Glycolic acid	CAS 79-14-1	Acros Organics	154510250
**DL-Lactic acid**	CAS 50-21-5	Acros Organics	125060250
Myristic acid	CAS 544-63-8	Acros Organics	156962500
Palmitic acid	CAS 57-10-3	Acros Organics	129702500
Pimelic acid	CAS 111-16-0	Acros Organics	131230250
Stearic acid	CAS 57-11-4	Alfa Aesar	A12244.06
trans-Aconitic acid	CAS 4023-65-8	Alfa Aesar	B20087.14
Mix 6			
Citric acid	CAS 77-92-9	Acros	A10395.30
**DL-Lactic acid**	CAS 50-21-5	Acros	125060250
L-Leucine	CAS 61-90-5	Acros	125121000
L-Phenylalanine	CAS 63-91-2	Acros	130310250
DL-Malic acid	CAS 6915-15-7	Acros	125252500
Succinic acid	CAS 110-15-6	Acros	219552500

Both standards were prepared in concentration ranges from **0.2 to 0.0000002** mg mL^{-1} and diluted 1:1 during silylation. Concentration of 0.002 was used to calculate the response of the detector for quantitative purposes. Mix10 was prepared and diluted in ethyl acetate, the solvent was evaporated in room temperature and the compounds were re-suspended in acetonitrile. To obtain Mix6, compounds were dissolved in acidic (~pH 2) ultrapure water, mixed, serially diluted, the solvent evaporated by freeze drying, with final re-suspension in acetonitrile to obtain the target concentrations.

Table S10 Compound parameters of the MRM transitions in the LC-SRM method developed for the analysis of amino acids in maize and tomato root exudates.

Q1 Mass (Da)	Q3 Mass (Da)	MRM transition name	Transition type	DP (volts)	CE (volts)	CXP (volts)
133.902	88.1	Aspartic acid-1	quantifier	11	13	10
133.902	74	Aspartic acid-2	qualifier	11	19	6
147.989	84	Glutamic acid-1	quantifier	11	21	8
147.989	130.1	Glutamic acid-2	qualifier	11	13	14
156.039	110	Histidine-1	quantifier	56	19	18
156.039	83.1	Histidine-2	qualifier	56	31	10
147.046	84.1	Lysine-1	quantifier	21	19	8
147.046	130	Lysine-2	qualifier	21	13	14
121.952	58.9	Cysteine-1	quantifier	16	31	12
121.952	76	Cysteine-2	qualifier	16	19	36
146.999	129.9	Glutamine-1	quantifier	51	13	16
146.999	84	Glutamine-2	qualifier	51	21	42
105.982	60	Serine-1	quantifier	1	15	10
105.982	87.9	Serine-2	qualifier	1	13	42
119.996	74	Threonine-1	quantifier	11	15	34
119.996	102.1	Threonine-2	qualifier	11	11	12
182.031	165.1	Tyrosine-1	quantifier	21	13	10
182.031	136	Tyrosine-2	qualifier	21	17	10
90.003	44.1	Alanine-1	quantifier	1	13	20
90.003	45	Alanine-2	qualifier	1	45	20
75.989	30	Glicyne-1	quantifier	21	21	14
75.989	48	Glicyne-2	qualifier	21	5	12
132.033	86	Isoleucine-1	quantifier	31	13	16
132.033	69.2	Isoleucine-2	qualifier	31	28	12
131.996	86	Leucine-1	quantifier	51	13	16
131.996	44.1	Leucine-2	qualifier	51	27	20
150.02	133	Methionine-1	quantifier	56	13	6
150.02	104.2	Methionine-2	qualifier	56	13	18
165.966	119.9	Phenylalanine-1	quantifier	41	17	16
165.966	102.9	Phenylalanine-2	qualifier	41	35	10
116.022	70.2	Proline-1	quantifier	1	19	8

116.022	43.1	Proline-2	qualifier	1	37	4
205.045	188	Tryptophan-1	quantifier	1	13	10
205.045	146.1	Tryptophan-2	qualifier	1	23	6
118.037	71.9	Valine-1	quantifier	41	15	10
118.037	55.2	Valine-2	qualifier	41	25	8
126	108	Taurine-1	quantifier	25	15	15
126	43.9	Taurine-2	qualifier	25	28	7
133.1	70	Ornithine-1	quantifier	10	25	7
133.1	116	Ornithine-2	qualifier	10	12.7	6
135.1	88.9	Leucine-D3-1	quantifier	15	13	11
135.1	45.3	Leucine-D3-2	qualifier	15	27	9
175.034	70.1	Arginine-1	quantifier	41	29	8
175.034	116.1	Arginine-2	qualifier	41	19	8

4 Supplementary Figures

Fig. S1 Growth of P482 in 1C medium supplemented with root exudates of maize or tomato. Panels A and B show growth in the presence of different concentrations of the root exudates of maize and tomato, respectively. For the RNAseq experiment, the cells were cultured in 0.2 mg L^{-1}. The OD_{600} of cultures was monitored in real time and the cells were collected after reaching early stationary phase per each condition (C).

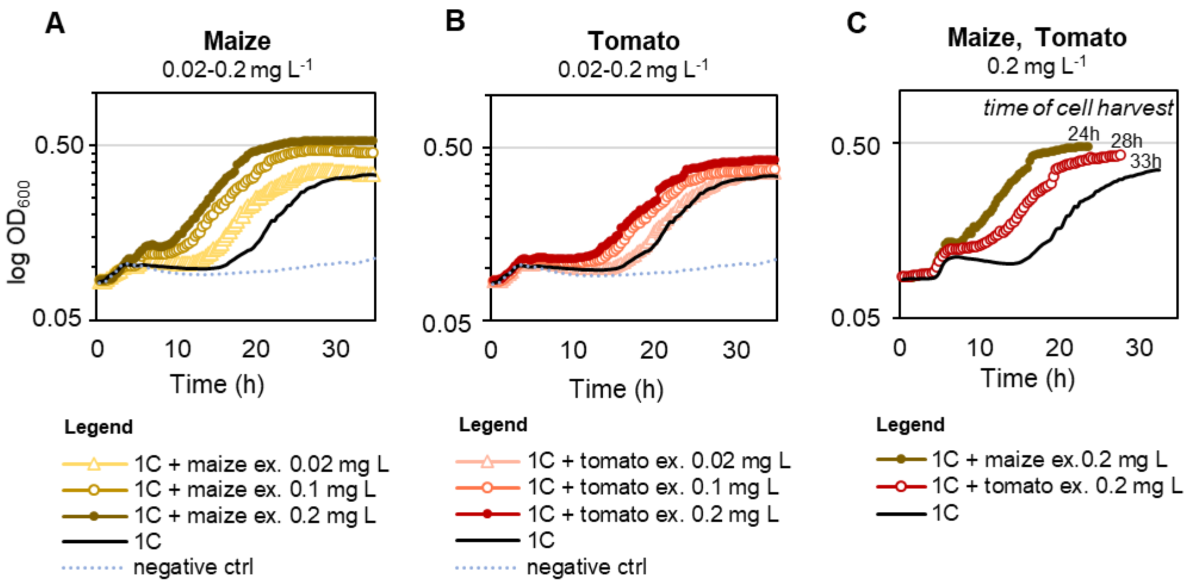

Fig. S2 Principal component analysis (PCA) plots depict overall transcriptome differences between samples. PCA assesses the variance in a dataset in terms of components defined on axes x and y. Data points correspond to RNA samples obtained for P482 grown in different experimental conditions: '1C' – unsupplemented 1C medium, '1C + tomato ex. 0.2 mg L^{-1}' – medium supplemented with tomato exudates and '1C + maize ex. 0.2 mg L^{-1}' medium supplemented with maize exudates. Panels A-C show: A – 1C vs 1C + tomato ex. 0.2 mg L^{-1}; B – 1C vs 1C + maize ex. 0.2 mg L^{-1}, C – 1C + tomato ex. 0.2 mg L^{-1} vs 1C + maize ex. 0.2 mg L^{-1}.

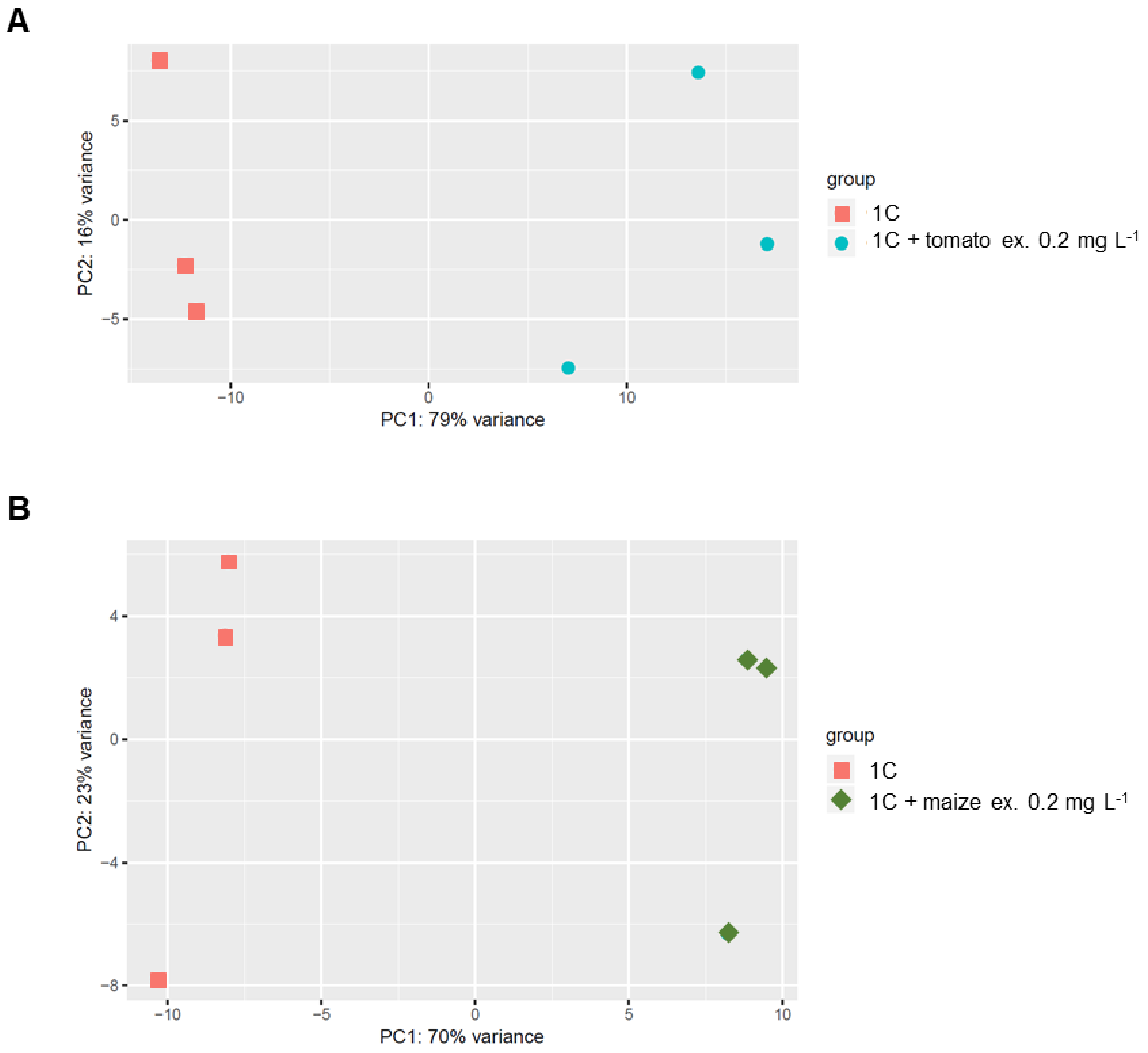

C

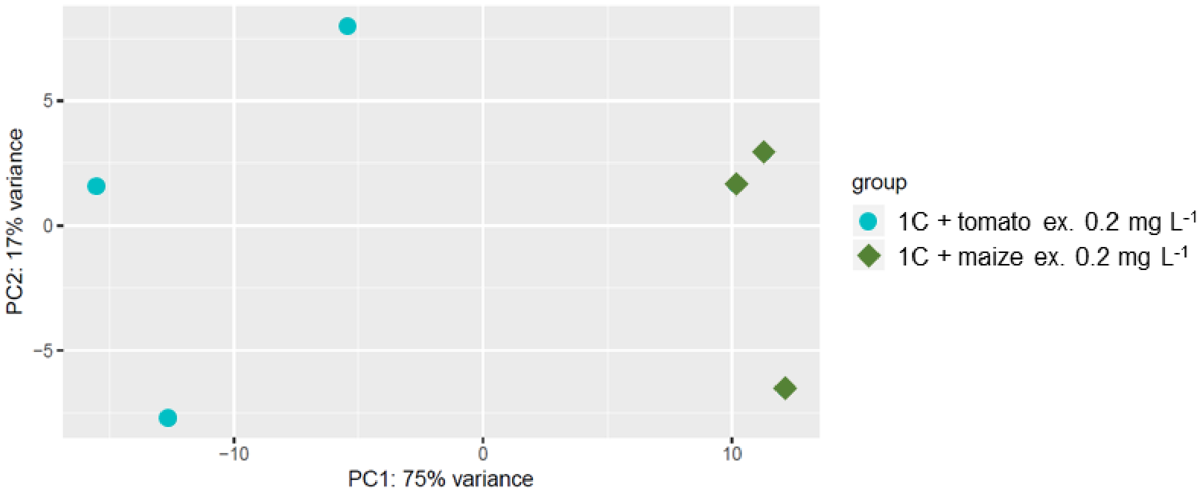
PC2: 17% variance
5
0
-5
-10
0
10
PC1: 75% variance
group
1C + tomato ex. 0.2 mg L-1
1C + maize ex. 0.2 mg L-1

Fig. S3 PCR products for the corresponding genes following electrophoresis in 1.7% agarose gel.

L – GeneRuler 100 bp Plus DNA Ladder

1 – *lrp* (113 bp)

2 – *mexE* (141 bp)

3 – *norC* (124 bp)

4 – *ssuC* (121 bp)

5 – *trpB* (125 bp)

6 – *ytfE* (120 bp)

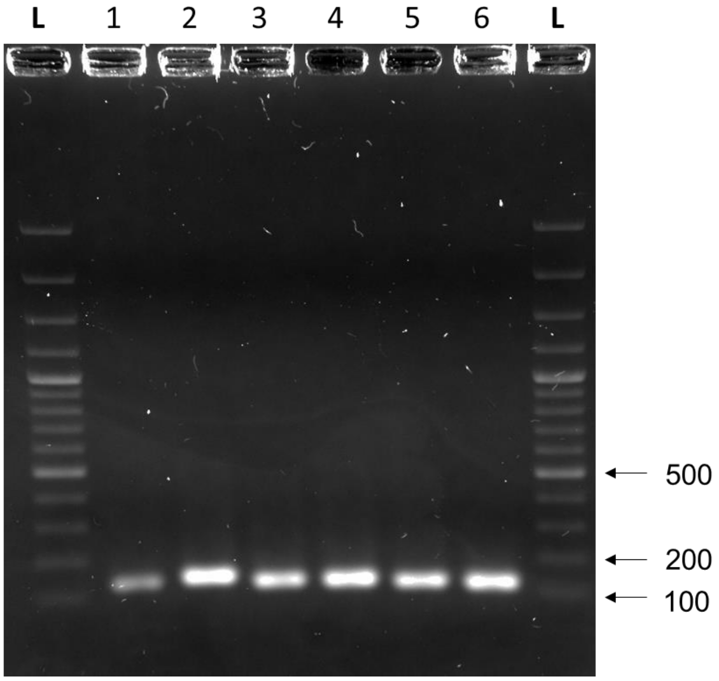

Fig. S4 The population size of *P. donghuensis* P482 Rif in the rhizosphere of soil-grown tomato and maize. Each data point represent the titer of cells per gram of rhizosphere sample (CFU g^{-1}). The '×' symbols represent averages. Horizontal lines represent median values. E1 and E2 stand for independent experiments, each including 7 replicates per species. The normality of data was determined using Shapiro-Wilk's test. Non-parametric two-tailed Mann–Whitney U test (α=0.05) was applied to determine the significance of differences between groups for data pooled from E1 and E2.

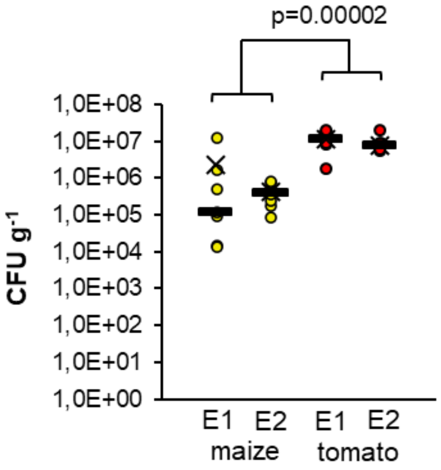

Fig. S5 Comparison of gene expression results obtained by RNAseq and RT-qPCR. The RT-qPCR experiment aimed to confirm the reliability of the RNAseq data. Analysis was performed for a group of genes representing both low and high differences in expression between treatments. Overall, the gene expression profile for all genes tested by RT-qPCR corresponded to the one obtained by RNAseq. For some genes like *ssuC* and *trpB* the results obtained by the two methods were near-identical. For *lrp*, for which the worst correlation could be claimed, the expression profile showed the same tendency of up- and-down regulation in the studied treatments, yet the absolute mean log_2FC values differed depending on the method (RNAseq vs RT-qPCR). The investigated targets included: *lrp* – putative transcriptional regulator (BV82_3254); *mexE* – efflux transporter, RND family, MFP subunit (BV82_2032); *norC* – nitric oxide reductase subunit C (BV82_3246); *ssuC* – binding--dependent transport system inner membrane component family protein (BV82_1676); *trpB* – tryptophan synthase, beta subunit (BV82_2326); *yftE* – hemerythrin HHE cation binding domain protein (BV82_3239). '1C + tom.' – P482 grown in 1C with the addition of tomato exudates; '1C + maize' – P482 grown in 1C with the addition of maize exudates. qPCR results were normalized to two reference genes: *gyrB* and *rpoD*. Stability of the applied reference genes was confirmed for the analyzed dataset (average M=0.543; CV=0.181). Expression was scaled to P482 not treated with exudates. Bars represent mean values for 3 biological replicates. Error bars show standard error of the mean (SEM). Stars indicate statistical significance of differences between groups ($p<0.05$), calculated with an unpaired t-test. Blue and red backgrounds indicate down- and up-regulation of gene expression, respectively.

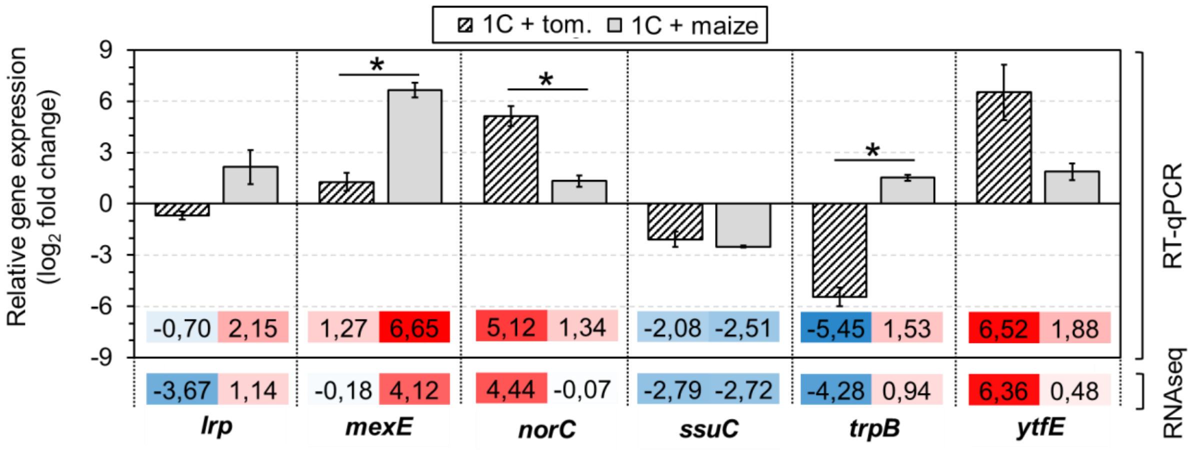

Fig. S6 Total ion chromatograms obtained by GC-MS for sylilated root exudates of maize cv. Bajm (A) and tomato cv. Saint Pierre (B). The compounds were identified based on mass spectra.

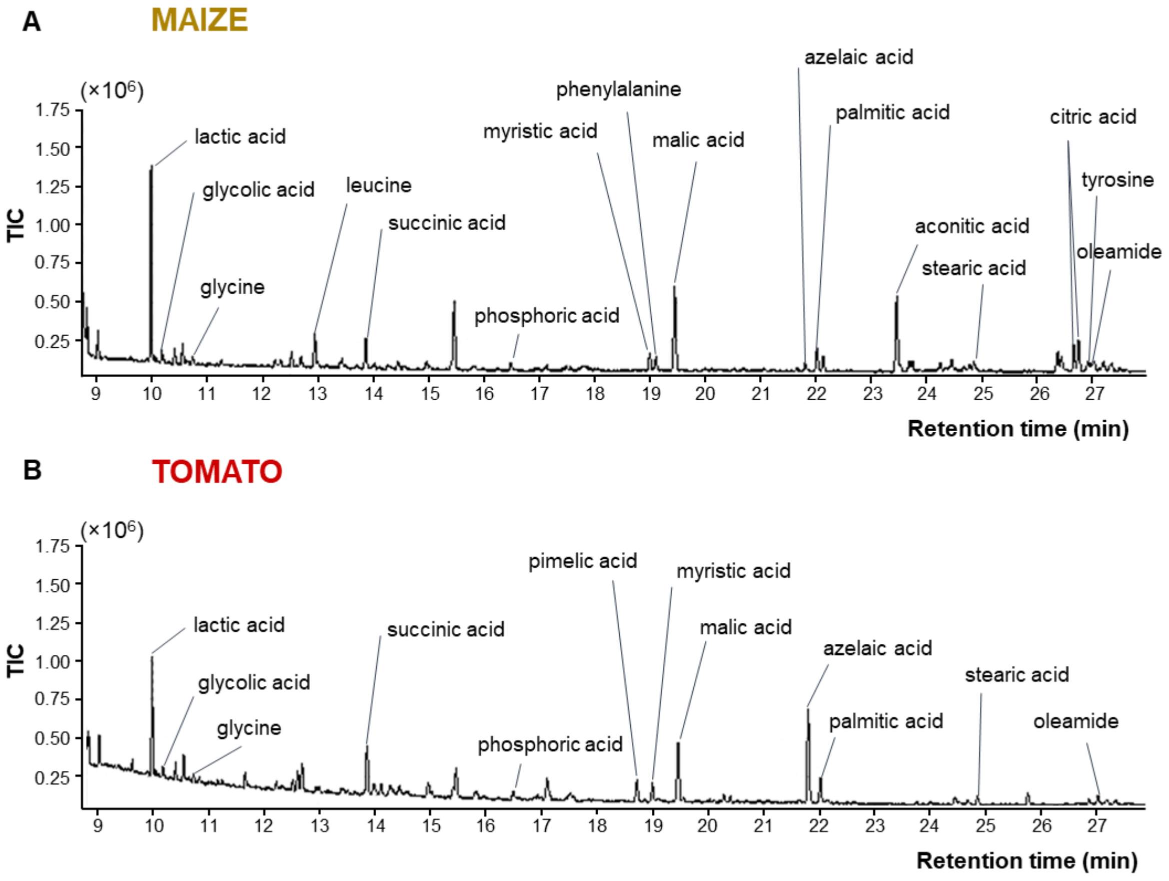

Fig. S7 1-D ^{1}H NMR spectra of maize (A) and tomato (B) root exudates.

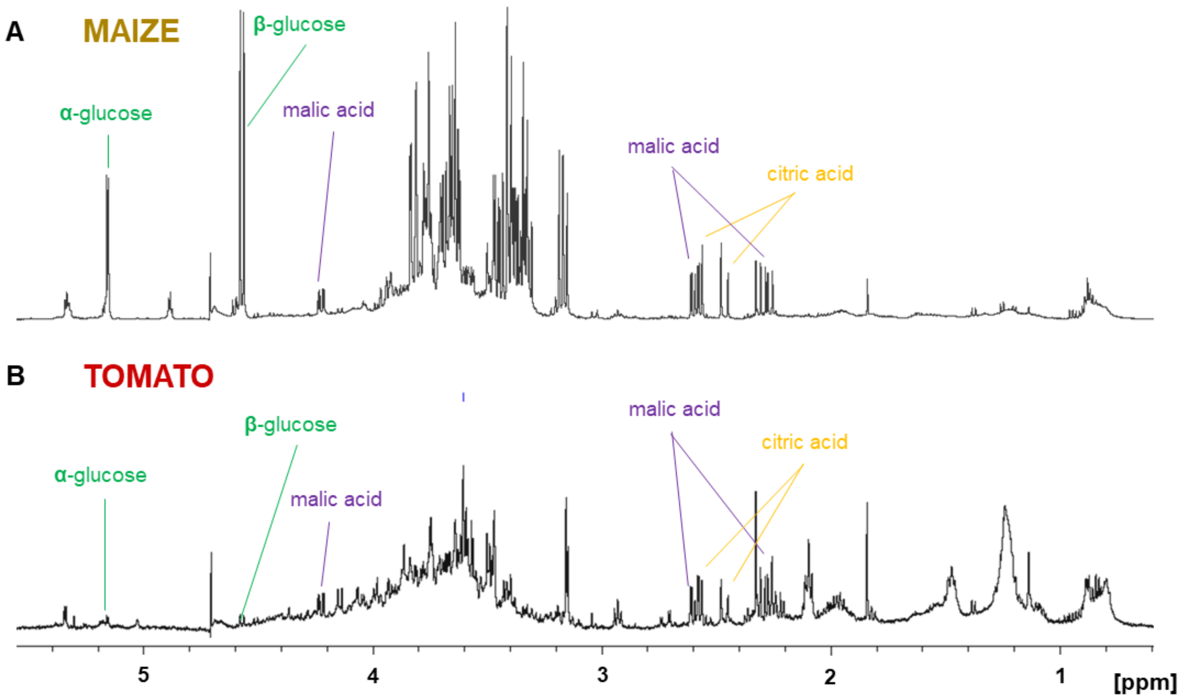

5 Supplementary References

1 Krzyzanowska, D. M. *et al.* Rhizosphere bacteria as potential biocontrol agents against soft rot caused by various *Pectobacterium* and *Dickeya* spp. strains. *Journal of Plant Pathology* **94**, 367-378 (2012).

2 Krzyzanowska, D. M. *et al.* When Genome-Based Approach Meets the "Old but Good": Revealing Genes Involved in the Antibacterial Activity of *Pseudomonas* sp. P482 against Soft Rot Pathogens. *Front Microbiol* **7**, 782, doi:10.3389/fmicb.2016.00782 (2016).

3 Krzyzanowska, D., Obuchowski, M., Bikowski, M., Rychlowski, M. & Jafra, S. Colonization of potato rhizosphere by GFP-tagged *Bacillus subtilis* MB73/2, *Pseudomonas* sp. P482 and *Ochrobactrum* sp. A44 shown on large sections of roots using enrichment sample preparation and confocal laser scanning microscopy. *Sensors (Basel)* **12**, 17608-17619, doi:10.3390/s121217608 (2012).

4 Maciag, T., Krzyzanowska, D. M., Jafra, S., Siwinska, J. & Czajkowski, R. The Great Five-an artificial bacterial consortium with antagonistic activity towards *Pectobacterium* spp. and *Dickeya* spp.: formulation, shelf life, and the ability to prevent soft rot of potato in storage. *Appl Microbiol Biotechnol* **104**, 4547-4561, doi:10.1007/s00253-020-10550-x (2020).

5 Fan, T. W. M., Lane, A. N., Pedler, J., Crowley, D. & Higashi, R. M. Comprehensive Analysis of Organic Ligands in Whole Root Exudates Using Nuclear Magnetic Resonance and Gas Chromatography–Mass Spectrometry. *Analytical Biochemistry* **251**, 57-68, doi:10.1006/ABIO.1997.2235 (1997).

6 Kudjordjie, E. N., Sapkota, R., Steffensen, S. K., Fomsgaard, I. S. & Nicolaisen, M. Maize synthesized benzoxazinoids affect the host associated microbiome. *Microbiome* **7**, 1-17 (2019).

7 Krzyżanowska, D. M., Iwanicki, A., Czajkowski, R. & Jafra, S. High-quality complete genome resource of tomato rhizosphere strain Pseudomonas donghuensis P482, a representative of a species with biocontrol activity against plant pathogens. *Molecular Plant-Microbe Interactions* **34**, 1450-1454 (2021).

8 Fortier, L. C. & Sekulovic, O. Importance of prophages to evolution and virulence of bacterial pathogens. *Virulence* **4**, 354-365, doi:10.4161/viru.24498 (2013).

9 Matuszewska, M., Maciag, T., Rajewska, M., Wierzbicka, A. & Jafra, S. The carbon source-dependent pattern of antimicrobial activity and gene expression in *Pseudomonas donghuensis* P482. *Sci Rep* **11**, 10994, doi:10.1038/s41598-021-90488-w (2021).
